# Multimodality Imaging of Mitral Regurgitation

**DOI:** 10.3390/jcm15197460

**Published:** 2026-09-25

**Authors:** Angelo Squeri, Valeria Maria De Luca, Stefano Censi, Carmine Pizzi, Luca Bergamaschi, Nicole Suma, Nicola Forzati, Rita Conti

**Affiliations:** 1 UO Cardiologia, IRCCS Maria Cecilia Hospital, Care & Research, 48033 Cotignola, Italy; 2 Department of Medical and Surgical Sciences (DIMEC), Alma Mater Studiorum—University of Bologna, 40138 Bologna, Italy; 3 Cardiovascular Division, Morgagni-Pierantoni University Hospital, 47121 Forlì, Italy

**Keywords:** mitral regurgitation, multimodality imaging, echocardiography, cardiac magnetic resonance, computed tomography

## Abstract

Mitral regurgitation is one of the most common valvular heart diseases and requires an accurate diagnostic approach to define its etiology, mechanism, severity, and therapeutic implications. This review analyzes the integrated role of the main cardiovascular imaging modalities in the assessment of mitral regurgitation. Transthoracic echocardiography (TTE) represents the first-line imaging modality, providing a dynamic assessment of valve anatomy, ventricular function, and hemodynamic consequences, whereas transesophageal echocardiography (TEE) provides complementary high-resolution anatomical characterization. Cardiac magnetic resonance provides accurate quantification of ventricular volumes, the regurgitant volume and fraction, and myocardial remodeling and is particularly useful when echocardiographic findings are inconclusive or discordant. Computed tomography, owing to its high spatial resolution, offers detailed information on the mitral apparatus, annulus, calcifications, and anatomical relationships, supporting the planning of surgical and transcatheter procedures. The integration of echocardiography, CMR, and CT enables more comprehensive risk stratification and personalized treatment selection. The review highlights how multimodality imaging is now essential for guiding the diagnostic and therapeutic pathway, improving intervention timing, the procedural choice, and the overall management of patients with mitral regurgitation.

## 1. Introduction

Mitral regurgitation (MR) is one of the most common valvular heart diseases, second only to aortic stenosis in prevalence [1,2].

The prevalence of moderate-to-severe MR is about 0.67%, but it increases sharply with age, reaching 2.85% in individuals over 70 and 6.45% in those over 90 [1]. As a result, MR is becoming a growing public health issue with significant clinical and social implications. Its impact is further highlighted by its high frequency among patients with left ventricular (LV) systolic dysfunction: up to 56.2% of those with chronic LV dysfunction have MR [3].

Despite its prevalence and prognostic relevance, up to half of patients with significant MR do not receive appropriate treatment [4]. Mitral regurgitation may remain asymptomatic for years or may present with acute heart failure.

Up to 44.5% of patients with acute heart failure have MR [5]. Even if the patient is asymptomatic, when MR is left untreated, it triggers a vicious cycle of progressive LV dilatation, persistent volume overload, increased wall stress, and further ventricular remodeling. Consequently, MR can lead to LV dysfunction, tricuspid regurgitation, pulmonary hypertension, atrial fibrillation, and death [6]. The severity of functional MR (FMR) is directly correlated with adverse outcomes. In an older cohort of patients with systolic heart failure, one-year mortality reached 45% and 57% in patients with grade moderate-to-severe (3+) and severe (4+) functional mitral regurgitation (FMR), respectively. Moreover, patients with both heart failure (HF) and FMR exhibit substantially higher mortality compared with those with HF alone [7,8].

From a diagnostic standpoint, transthoracic echocardiography has traditionally been the first-line method for MR evaluation. However, advanced imaging modalities have increasingly become part of routine clinical practice to fully analyze the MR mechanism and severity. In this regard, the combination of echocardiography, computed tomography (CT), and cardiac magnetic resonance (CMR) allows for the accurate characterization of valve anatomy and function, thereby guiding optimal treatment selection [9].

This review aims to emphasize the potential of multimodality imaging for the correct diagnosis of mitral regurgitation to facilitate the timely referral of patients to specialized heart valve centers. A narrative literature review was conducted to provide a comprehensive overview of the current role of multimodality imaging in the assessment and management of MR. Relevant literature was identified through a search of the PubMed/MEDLINE database, covering publications from 1994 to 2026. Particular emphasis was placed on contemporary international guidelines, scientific statements, consensus and position papers, and key prospective and observational studies. Publications addressing the diagnostic, prognostic, and therapeutic implications of transthoracic and transesophageal echocardiography, cardiac magnetic resonance, and cardiac computed tomography were considered. Particular attention was given to studies reporting quantitative imaging parameters, diagnostic thresholds, and outcome-related measures relevant to MR severity assessment and patient management.

## 2. Anatomy and Pathophysiology of Mitral Regurgitation

The mitral valve is a complex structure composed of the anterior and posterior leaflets, the mitral annulus (MA), the subvalvular apparatus (including the chordae tendineae and papillary muscles), and the left ventricle. Any pathological process involving one or more of these components can lead to MR. As a result, the assessment of mitral valve function, severity, and mechanism can be challenging [7,8].

For many years, the Carpentier classification has been the cornerstone for evaluating the mechanisms of MR and remains clinically valuable. However, in numerous patients, multiple mechanisms may coexist and contribute to the development of MR.

The most clinically relevant and prognostically meaningful distinction is between primary and secondary mitral regurgitation. MR exhibits substantial heterogeneity in its pathophysiology depending on whether the underlying mechanism is classified as primary (organic) or secondary (functional) [9].

### 2.1. Primary Mitral Regurgitation (Organic)

Primary or organic MR refers to a group of congenital and acquired disorders that cause intrinsic structural abnormalities of the mitral valve leaflets or the subvalvular apparatus. Typical etiologies include mitral valve prolapse, flail leaflet, myxomatous degeneration, and rheumatic disease.

Mitral valve prolapse (MVP) represents the most common underlying cause of organic MR; therefore, primary MR is most commonly associated with Carpentier type II dysfunction. However, type I dysfunction may occur in the context of endocarditis, while type IIIa is observed in rheumatic heart disease [9]. Primary mitral regurgitation represents the group of conditions most frequently managed surgically [10]. MVP encompasses a broad spectrum of pathologies, ranging from isolated scallop prolapse to complex multiscallop involvement. These lesions span from fibro-elastic deficiency, which is more common in older patients, to milder forms and fruste pathologies, up to Barlow’s disease, characterized by marked bileaflet and multiscallop prolapse, annular dilatation, leaflet thickening, excess leaflets tissue, chordal elongation, or rupture [10].

In patients with mitral valve prolapse, the five-year cardiovascular mortality is approximately 9%, increasing to 19% when all-cause mortality is considered [11]. About 8% of these patients eventually require surgery, and up to 20% develop complications such as heart failure. The slow remodeling of the left ventricle in response to chronic volume overload may render the disease clinically silent for years. However, progressive dilation is associated with left ventricular dysfunction and the onset of symptoms, including dyspnea, shortness of breath, orthopnea, and paroxysmal nocturnal dyspnea. Without surgical intervention, patients with chronic primary MR have an annual mortality rate of about 6% [11]. Therefore, accurate diagnosis and early risk stratification are essential for guiding prognosis and treatment. Among the most widely used tools for this purpose is the MIDA-Q score mortality index. The MIDA-Q mortality index incorporates the variables included in the original MIDA score together with additional parameters derived from an echocardiographic assessment of the effective regurgitant orifice area (EROA) [12,13]. Specifically, it includes clinical variables such as age ≥65 years (3 points), New York Heart Association (NYHA) class ≥ III (3 points), and atrial fibrillation (1 point), as well as echocardiographic variables including Left Atrial Volume Index (LAVI) ≥ 60 mL/m^2^ or left atrial diameter ≥55 mm (1 point), pulmonary artery systolic pressure ≥50 mmHg (2 points), left ventricular end-systolic diameter ≥ 40 mm (1 point), left ventricular ejection fraction <60% (1 point), and EROA 20–40 mm^2^ (1 point), 40–60 mm^2^ (2 points), or >60 mm^2^ (3 points). Five-year mortality ranged from 3% in patients with a MIDA-Q score of 0 to 95% in those with a score ≥ 13 [13]. Therefore, calculating the MIDA-Q score after the echocardiographic diagnosis of MVP is a useful tool to identify higher-risk patients and guide them toward prompt intervention.

### 2.2. Secondary Mitral Regurgitation (Functional)

Secondary or ventricular functional MR (vFMR) arises from left ventricular remodeling or dysfunction, leading to papillary muscle displacement, leaflet tethering, and impaired coaptation in the absence of primary leaflet or chordal pathology; concomitant annular dilatation may also occur.

vFMR is highly prevalent in patients with heart failure and is associated with worse outcomes due to ongoing volume overload and progressive adverse ventricular remodeling.

In patients with vFMR, leaflet tethering may be either symmetric or asymmetric (Carpentier IIIB mechanism). Asymmetric tethering of the posterior mitral leaflet typically produces an eccentric or commissural jet. This pattern is observed in ischemic heart disease or following a posterior myocardial infarction, where displacement of the posterior papillary muscle occurs. When the left ventricle is globally dilated—as after an anterior infarction or in nonischemic cardiomyopathy—both papillary muscles are displaced, resulting in symmetric tethering and a predominantly central regurgitant jet.

In addition, MR may result from isolated annular dilation (Carpentier I mechanism), typically observed in patients with heart failure with preserved ejection fraction and atrial fibrillation, a pattern referred to as atrial functional MR (aFMR). Annular dilatation is described if the ratio annulus diameter/anterior leaflet length is >1.3 (in diastole) or if the anteroposterior annulus diameter is >35 mm [14]. Nevertheless, these mechanistic categories often overlap, leading to mixed phenotypes that can further complicate the diagnostic assessment (dual FMR) [15].

Two phenotypes of aFMR can be identified. The more common presentation features a central regurgitant jet, resulting from impaired leaflet coaptation. In a smaller subset of patients (approximately 20–30%), the regurgitation is eccentric and directed posteriorly, caused by “hamstringing” of the posterior mitral leaflet. Progressive annular enlargement may subsequently lead to anterior leaflet override, as marked left atrial dilation—particularly posterior dilation—displaces the posterior MA beyond the crest of the left ventricular inflow tract. Because the left ventricle and sub valvular apparatus remain structurally normal, this pattern of leaflet tethering is referred to as atriogenic tethering [16].

Compared to vFMR patients, aFMR patients have a decreased rate of HF hospitalization and all-cause mortality.

Every year, 24% of patients with vFMR and 10% of patients with aFMR advanced to dual FMR. Dual FMR has the highest incidence of all-cause mortality and HF hospitalization among all three categories [15,17].

## 3. Overview of Imaging Modalities

A comprehensive assessment of mitral regurgitation requires a careful evaluation of several aspects: (1) multiparametric quantification of the severity of regurgitation; (2) a thorough assessment of the anatomy and function of the mitral valve complex, including the annulus, leaflets, and subvalvular apparatus, in order to identify lesions or dysfunctions responsible for mitral regurgitation; and (3) quantification of left ventricular and left atrial dimensions, as well as pulmonary artery pressures and right ventricular function.

Nowadays, the different imaging modalities available allow us to assess all of these aspects. However, each technique has its own advantages and limitations, which must be well understood in order to achieve an accurate diagnosis and a correct interpretation of the clinical picture.

### 3.1. Transthoracic Echocardiography (TTE)

Echocardiography is the mainstay for non-invasive MR quantification, providing versatile tools for both qualitative and quantitative analysis. Transthoracic echocardiography (TTE) is the first-line diagnostic modality for evaluating MR severity owing to its accessibility and non-invasive nature. Despite its widespread use, the technique faces challenges in precisely quantifying the regurgitant jets, particularly when imaging quality is suboptimal [9].

Even though it has some limitations, TTE remains the best imaging technique for MR evaluation. TTE, in addition to being a non-invasive and widely available examination, allows for a simultaneous anatomical and functional assessment of the mitral valve, accurate quantification of the severity of regurgitation, and evaluation of the biventricular and atrial size and function.

In particular, it allows an estimation of forward flow, analysis of the regurgitant mechanism, and quantification of the regurgitant volume (RVol), regurgitant fraction (RF), and EROA [9].

During TTE, a complete analysis of mitral valve apparatus is also important. An evaluation of the leaflets, annulus, and subvalvular apparatus is essential for understanding the mechanism of regurgitation and thus assessing its etiology.

A comprehensive evaluation of mitral anatomy and function must follow a complete standardized acquisition protocol (Figure 1).

### 3.2. Transesophageal Echocardiography (TEE)

TEE is a semi-invasive imaging technique that provides a high-resolution assessment of mitral valve anatomy and function. Compared with TTE, TEE provides higher spatial resolution, particularly for posterior cardiac structures and for the detailed assessment of mitral valve morphology, mainly because of the closer proximity of the transducer to the valve and the use of higher ultrasound frequencies.

Moreover, when surgical or percutaneous treatment is being considered, TEE is essential for a precise anatomical evaluation and a more detailed scallop-by-scallop analysis. A standardized protocol, similar to that used for transthoracic echocardiography, is necessary for a comprehensive anatomical assessment and should include multiple 2D, 3D, and Doppler acquisitions to ensure an accurate evaluation of mitral regurgitation (Figure 2). Particular attention should be paid to obtaining appropriate, non-off-axis views, thereby enabling an assessment of the lesions and the spatial relationships among the different cardiac structures.

As a semi-invasive procedure that may cause patient discomfort, TEE often requires mild sedation to improve comfort and facilitate the acquisition of high-quality images. However, sedation can alter loading conditions and may therefore lead to an underestimation of mitral regurgitation severity. This effect is particularly relevant when TEE is performed under general anesthesia, such as in the cardiac operating room or during structural heart interventions, where anesthesia-related hemodynamic changes may further reduce the apparent severity of mitral regurgitation.

### 3.3. Cardiac Magnetic Resonance (CMR)

Although echocardiography remains the primary imaging modality for the evaluation and assessment of MR, CMR has emerged as a robust secondary investigation, offering both quantitative and qualitative assessments of mitral valve disease.

CMR offers several advantages and is particularly useful in selected clinical scenarios, such as in the presence of suboptimal echocardiographic windows (e.g., due to body habitus, pectus excavatum, or obesity) [18], eccentric valvular lesions, multiple regurgitant jets, and right-sided valvular disease. Furthermore, CMR is widely regarded as the reference standard for quantifying ventricular and atrial volumes, which is particularly important because mitral regurgitation has hemodynamic consequences for the size and function of the left atrium (LA) and left ventricle (LV); it also provides comprehensive tissue characterization [19,20].

CMR may be contraindicated in the presence of certain non-MR-compatible ferromagnetic devices or foreign bodies and may be challenging in patients with severe claustrophobia. Renal function should be considered when gadolinium-based contrast administration is required. In addition, arrhythmias and susceptibility artifacts from implanted cardiac devices may impair image quality [21].

CMR is also a time-consuming examination that requires substantial patient compliance and the availability of appropriately trained medical personnel.

A comprehensive valve imaging protocol typically begins with standard long-axis cine acquisitions in the two-, three-, and four-chamber views to evaluate mitral valve disease. A short-axis cine stack with a slice thickness of 5 mm and no gap and a temporal resolution of ≤45 ms from the atrioventricular plane to the apex is then obtained to delineate valvular anatomy and motion, as well as to quantify left and right ventricular volumes, stroke volumes, and ejection fractions in systole and diastole. Phase-contrast (PC) flow imaging represents a crucial component of the protocol, enabling the direct quantification of flow and velocity in regurgitant and stenotic lesions, respectively. In routine practice, phase-contrast sequences are acquired perpendicular to the aortic root and main pulmonary artery. In patients with arrhythmias or impaired breath-holding capacity, free-breathing acquisitions with increased signal averaging are required to ensure adequate image quality [14].

Beyond the standard protocol, T1 mapping techniques and inversion recovery sequences for late gadolinium enhancement may be integrated to identify myocardial fibrosis and the scar burden. The presence and extent of myocardial fibrosis and scars have been demonstrated to add prognostic value, especially in patients with mitral valve prolapse, allowing the identification of patients at an increased risk of arrhythmias and adverse cardiovascular events [22].

### 3.4. Cardiac Computed Tomography

For the evaluation of cardiac valve anatomy and function, 64-slice or more advanced technology such as dual source CT or volume CT scanners is required. ECG gating, including the acquisition of a multiphase CT dataset covering the entire cardiac cycle, is fundamental for dynamic imaging. Retrospective ECG-gated spiral scanning is therefore the suggested acquisition mode, obtaining data throughout the complete cardiac cycle [23].

In order to obtain systolic and diastolic views of the MV and 4D cine loops for a functional assessment, image reconstruction of axial thin slice source images of less than 1 mm at either 5 or 10% increments of the RR interval is recommended.

The same data are also used for LV function analysis.

Intravenous iodinated contrast agent, at a rate of 3–5 mL/s, should be injected to visualize cardiac and vascular structures. The timing of the contrast agent injection can be done via a test bolus or via bolus triggering to obtain a good opacification of the left heart chambers [24].

CT imaging for mitral valve assessment is generally considered a safe procedure.

Nevertheless several aspects should be taken into account, including radiation exposure and potential effects of iodinated contrast media administration.

The dose of ionizing radiation imparted to patients should be minimized according to the ALARA (“As low as reasonably achievable”) guidance for radiation safety.

For this purpose, it is advisable to limit the scan range, avoiding unnecessary extra coverage. Tube voltage should be set to the lowest level compatible with the patient’s body habitus and automatic current tube modulation should be applied during acquisition [25]. Prospective ECG-triggered acquisition may further decrease radiation exposure, as the scanner delivers radiation only during a predefined portion of the cardiac cycle, triggered by ECG, but it could be appropriate only when patients have a stable sinus rhythm with minimal R-R variability or when the clinical question is restricted to static features, such as mitral annular calcification or mitral annular morphology.

In patients with renal impairment, strategies to minimize the contrast volume are mandatory. These may include the use of high-iodine-concentration agents, allowing a reduction in the total contrast volume while maintaining adequate iodine delivery. A bolus tracking method should be preferred over an additional test bolus. When suitable for the patient’s body habitus, a low-tube voltage acquisition protocol can also improve iodine attenuation and allow a further contrast volume reduction.

A history of previous severe allergic reaction to iodinated contrast media generally supports an alternative imaging technique. If CT is essential, potential strategies include the use of a different contrast agent, appropriate premedication, and performing the study in equipped settings with anaphylaxis management available [26].

Another fundamental issue is image quality, which may be significantly affected by the cardiac rhythm and patient-related factors. Specifically, atrial fibrillation, frequent ectopy, a high heart rate (which are very common in patients affected by MR), severe obesity, or the inability to cooperate may produce nondiagnostic images.

In similar situations, retrospective ECG-gated acquisition, ECG trace editing (by excluding premature beats or very short R-R intervals), or absolute time-delay reconstructions in milliseconds are recommended.

Furthermore, intravenous beta-blocker administration is suggested if the heart rate is over 70 bpm.

Post-processing requires dedicated advanced workstations equipped with multiplanar reformations (MPRs) for the generation of valvular planes, a volume rendering technique (VRT) for visualizations of leaflet full anatomy, and maximum intensity projections (MIP). Finally, the generation of 4D cine imaging video loops during the entire cardiac cycle allows for an evaluation of leaflet function.

## 4. Quantification of Mitral Regurgitation

Current evidence supports a standardized grading of MR on a four-level scale (mild, moderate, moderate-to-severe, and severe), integrating qualitative, semi-quantitative, and quantitative echocardiographic criteria (Table 1) [14,27]. The assessment of MR severity should rely on a multiparametric approach to reduce interobserver variability.

The parameters used to quantify the severity of mitral regurgitation can be divided into qualitative, semiquantitative, and quantitative parameters.

Mitral regurgitation is generally considered severe when the effective regurgitant orifice area (EROA) is ≥40 mm^2^ and/or the regurgitant volume (RVol) is ≥60 mL, with a regurgitant fraction ≥ 50% providing additional support for severity grading. In secondary mitral regurgitation (SMR), an assessment should be performed after the optimization of guideline-directed medical therapy and under stable, euvolemic, and normotensive conditions. Because low-flow states and the frequently elliptical shape of the regurgitant orifice may result in lower EROA and RVol values, an EROA ≥ 30 mm^2^ and/or an RVol ≥ 45 mL may identify clinically significant SMR. Even lower values (EROA ≥ 20 mm^2^ and/or RVol ≥ 30 mL) have been associated with adverse outcomes, but should not be used in isolation to define severe SMR or to guide intervention [18].

### 4.1. Echocardiography

The assessment of mitral regurgitation severity begins with a careful visual evaluation of the origin, number, and location of the regurgitant jets, followed by a comprehensive multiparametric assessment using qualitative, semiquantitative, and quantitative parameters. Finally, the hemodynamic impact of mitral regurgitation is evaluated by assessing the left ventricular and left atrial size and function as markers of volume overload.

Continuous-wave Doppler signal density is considered a qualitative parameter for the assessment of mitral regurgitation, whereas color flow Doppler imaging, the vena contracta width, the anterograde velocity of mitral inflow, the mitral to aortic VT ratio, and a pulmonary vein flow assessment are regarded as semiquantitative methods.

The quantitative assessment of mitral regurgitation is based on direct measurement of the effective regurgitant orifice area (EROA), the regurgitant volume, and the regurgitant fraction, typically derived using the PISA method [27]. No single parameter is sufficiently robust across all clinical and hemodynamic conditions. The interpretation of individual parameters should take into account jet morphology, loading conditions, the dynamic nature of the regurgitant orifice, the presence of multiple regurgitant jets, and the duration of MR throughout systole [27]. Importantly, the quantitative thresholds reported below should always be interpreted according to the underlying mechanism of MR. The severity criteria and prognostic implications of quantitative parameters may differ among primary MR, vFMR, and aFMR.

#### 4.1.1. Color Flow Doppler Imaging

Color flow Doppler imaging is widely used for the initial assessment of mitral regurgitation, as it allows rapid identification of the origin and direction of the regurgitant jet and provides useful information regarding the underlying mechanism of MR. However, because the color Doppler appearance is strongly influenced by technical settings, jet direction, driving pressure, and hemodynamic conditions, the jet area should not be used as a standalone parameter for grading MR severity [14].

Although a larger color Doppler jet may suggest a greater regurgitant volume, the relationship between the jet area and MR severity is highly variable. Jet size is influenced by several factors, including the jet direction, the pressure gradient between the left ventricle and left atrium, echocardiographic settings, and left atrial compliance. Accordingly, relatively small jets may occur despite substantial regurgitant volumes. This is particularly relevant in acute MR, in which reduced left atrial compliance and elevated left atrial pressure may limit jet expansion within the atrium.

Eccentric regurgitant jets, such as those associated with leaflet prolapse, flail leaflet, or calcific valve disease, may also lead to the underestimation of MR severity via color Doppler imaging. In these settings, the “splay” artifact may further complicate visual assessments. Therefore, when TTE findings are inconclusive or discordant with other parameters of MR severity, further evaluation with TEE should be considered [14].

#### 4.1.2. Anterograde Velocity of Mitral Inflow and Mitral to Aortic Velocity Ratio

In the presence of significant mitral regurgitation, the increased transmitral flow results in higher early diastolic filling velocities and, consequently, an increased E-wave velocity. Therefore, in the absence of mitral stenosis, an E-wave velocity > 1.2 m/s is suggestive of severe mitral regurgitation. Conversely, a transmitral filling pattern with A-wave predominance makes severe mitral regurgitation unlikely.

The discrepancy between transmitral inflow and transaortic forward flow provides a semiquantitative estimate of the volume of mitral regurgitation. This can be assessed using the Pulsed Doppler (PW) mitral inflow-to-LVOT VTI ratio, calculated by tracing the velocity–time integral (VTI) of mitral inflow at the level of the mitral leaflet tips and the VTI in the left ventricular outflow tract (LVOT). A VTI ratio > 1.4 is highly suggestive of severe mitral regurgitation.

#### 4.1.3. Pulmonary Venous Flow

In healthy individuals, pulmonary vein flow is characterized by a positive systolic S wave, followed by a smaller diastolic D wave and, subsequently, by the atrial reversal wave. In the presence of significant mitral regurgitation, the systolic S-wave velocity progressively decreases and may eventually become completely reversed. Several conditions, including diastolic dysfunction and atrial fibrillation, may also result in reduced S-wave velocity. However, systolic flow reversal in the pulmonary veins is one of the strongest and most specific findings supporting the diagnosis of severe mitral regurgitation.

Pulmonary vein flow can be assessed using pulsed-wave Doppler, with the sample volume positioned 10 mm from the pulmonary vein ostium. With transthoracic echocardiography, the right upper pulmonary vein is generally the easiest to sample, whereas during transesophageal echocardiography, it is important to assess all pulmonary veins, particularly in the presence of mitral regurgitation with markedly eccentric jets.

#### 4.1.4. Vena Contracta

Vena contracta (VC) represents the narrowest portion of the regurgitant jet just downstream of the regurgitant orifice and it represents a surrogate measurement of the regurgitant orifice. The vena contracta width should be measured only when all three components of the regurgitant jet—the flow convergence region, the vena contracta, and the distal jet—are clearly visualized. This method is based on the assumption that the regurgitant orifice is circular. This is usually true in PMR. On the contrary, in FMR, the regurgitant orifice is usually elliptical or slit-like, suggesting the measurement of VC averaging 2–3 cardiac cycles using two orthogonal planes. A mean value > 8 mm is reported to correlate with severe mitral regurgitation. However, in the presence of multiple regurgitant jets, the vena contracta widths of individual jets should not be summed, as this approach is not validated and may lead to an inaccurate estimation of overall regurgitation severity [14].

#### 4.1.5. PISA

The PISA method allows a calculation of the EROA and regurgitant volume (RVol) from the proximal flow convergence region located on the ventricular side of the mitral valve, upstream of the regurgitant orifice during systole. Limitations include geometric assumptions, particularly in eccentric jets, with differences between FMR and PMR. Inaccuracies in MR quantification are also due to suboptimal alignment.

EROA and RVol are most commonly calculated using the PISA method. Volumetric quantification, either using the estimated flow through the aortic and MA or through the difference between the absolute stroke volume and forward stroke volume is especially relevant in FMR, where geometric assumptions linked to the PISA method may fail due to slit-like orifices.

The apical four-chamber, two-chamber, or three-chamber views are usually recommended for optimal visualization of the PISA. To improve image quality and visualization of PISA, the Nyquist limit is set to 15–40 cm/s and image depth is reduced.

Great attention must be paid to the acquisition of the CW Doppler signal of mitral regurgitation, especially in a very eccentric jet where maximum jet velocity can be underestimated, leading to a falsely larger EROA measurement. In non-holosystolic MR, particularly when the regurgitant flow varies substantially during systole, measurements obtained at a single time point may not accurately represent the total regurgitant burden.

It should be noted that the PISA radius may vary during systole due to the mitral regurgitation mechanism. FMR usually tends to have a larger PISA radius in early and late systole, while PMR has a larger PISA radius in late systole. Primary MR is severe when EROA is ≥0.4 cm^2^ with a regurgitant volume greater than 60 mL/beat and a regurgitant fraction more than 50% [14]. In FMR, lower EROA and RVol values may carry prognostic significance; therefore, quantitative PISA-derived parameters should be interpreted within an integrated multiparametric assessment, taking into account the loading conditions, regurgitant orifice geometry, and overall forward stroke volume [28,29].

#### 4.1.6. Volumetric Method

In the volumetric method, the total stroke volume (TSV) is calculated from the difference between left ventricular end-diastolic volume (LVEDV) and left ventricular end-systolic volume (LVESV). The effective forward stroke volume is then obtained using the Doppler approach, by multiplying the LVOT area by the velocity–time integral (VTI) of the LVOT (Figure 3). This method provides a quantitative estimate of mitral regurgitation severity by comparing the total left ventricular stroke volume with the effective forward flow, thereby allowing a calculation of the regurgitant volume and regurgitant fraction. Although conceptually robust, the volumetric method is sensitive to measurement errors in ventricular volumes and LVOT dimensions and should be integrated with other qualitative and semi-quantitative parameters within a multiparametric assessment strategy. Another potential source of error is the underestimation of LVEDV especially using Simpson’s method due to apical foreshortening. Nowadays, the introduction of a rapid 3D assessment of LV volumes and function partially overcomes these limitations. In addition, this method can be used in the absence of significant aortic regurgitation.

Given the limitations described above, no single parameter is sufficient on its own for the diagnosis and grading of MR. Instead, all available findings should be carefully integrated taking into account the strengths and limitations of each method. In addition, the assessment of left atrial and left ventricular remodeling should be systematically incorporated into the diagnostic work-up of MR, as it provides important complementary information regarding disease severity, chronicity, and clinical impact.

### 4.2. CMR

The quantification of MR can be either qualitative or quantitative.

#### 4.2.1. Qualitative Assessment

The regurgitant flow can be assessed using both cine b-SSFP and phase-contrast sequences. In cine images, flow visualization relies on signal loss caused by turbulent flow, and long-axis views allow an evaluation of the jet origin and direction (central versus eccentric), as well as distinction among early-, mid-, or holosystolic jets. However, when the regurgitant jet is broad and laminar, as occurs with large orifice areas, signal loss may be minimal, potentially resulting in an underestimation of severe regurgitation [30]. Representative cine b-SSFP images of secondary MR are shown in Figure 4.

#### 4.2.2. Quantitative Assessment

Quantitative techniques are considered the preferred approach for CMR evaluation of mitral RVol, although CMR-derived thresholds should not be considered interchangeable with guideline-based echocardiographic cut-offs.

RVol can be directly assessed using short-axis, through-plane 2D-PC sequences perpendicular to the MR jet, but this may underestimate eccentric or multiple jets. Indirect quantification is therefore the preferred approach for measuring RVol and RF.

The standard method for calculating RVol consists of subtracting aortic flow measured based on PC sequences from the left ventricular stroke volume (LVSV), which is derived from short-axis SSFP cine images. For LVSV quantification, the endocardial border of the left ventricle is delineated manually or semi-automatically on each short-axis slice at end-diastole and end-systole to determine end-diastolic and end-systolic volumes, with LVSV calculated as their difference. This approach is widely regarded as the most accurate and reproducible method for assessing R Vol [30]. This indirect quantification workflow is illustrated in Figure 5.

When aortic regurgitation (AR) is present, it must be taken into account. AR can be quantified directly from aortic diastolic flow mapping, and mitral regurgitant volume (RVol) is then calculated as LVSV minus the sum of aortic net forward flow and AR volume.

In the absence of intracardiac shunts, pulmonary flow measured via PC imaging can substitute for aortic flow, and when mitral regurgitation is the only valvular lesion, the right ventricular stroke volume (RVSV) may be used instead.

RF is thus expressed as the ratio of RVol to the left ventricular stroke volume (LVSV) multiplied by 100.

The 2022 EACVI position paper proposed the same RF thresholds for cardiac MRI as those established for echocardiography (mild regurgitation if RF < 30%, severe if RF ≥ 50%). However, the validity of these values requires further validation through the collection of additional prospective data [14].

The indirect volumetric approach allows an assessment of mitral regurgitation severity regardless of the number or direction of regurgitant jets and does not rely on geometric assumptions or the use of contrast agents [31].

It is important to acknowledge that the accurate CMR assessment of stroke volume and flow requires experienced operators and routine internal validation, since errors can arise from LV segmentation, inadequate breath-holding, arrhythmias, or the incorrect selection of velocity encoding (VENC)—particularly in the presence of high-velocity mitral regurgitation jets or concomitant valve stenosis.

#### 4.2.3. Four-Dimensional (4D) Flow Assessment

Four-dimensional flow is an emerging CMR technique that allows a direct assessment of mitral regurgitation and remains applicable in the presence of multiple valvular lesions or shunts [32].

Recommended acquisitions include a single acquisition, single sequence, and retrospectively ECG-gated sequences covering the full cardiac cycle with temporal resolution ≤ 45 ms and spatial resolution of approximately 3 × 3 × 3 mm [33].

Despite its promise in enhancing CMR evaluations, long acquisition times and demanding post-processing currently limit widespread clinical adoption [34].

#### 4.2.4. CMR vs. Echocardiography

Several studies (Table 2) have compared echocardiography and CMR for the evaluation of valvular regurgitation [20,35,36].

For MR, overall agreement between the two modalities is generally modest. Uretsky et al. reported that only 52% of cases classified as severe MR with 2D echocardiography were confirmed via CMR, with discordance particularly pronounced in patients with severe or secondary MR [42].

The widespread adoption of 3D echocardiography appears to improve concordance, as the 3D assessment of ventricular volumes has been shown to surpass 2D echocardiography, with overall agreement for severe MR ranging from 49% to 79% when compared with CMR [43,44].

In fact, in a recent meta-analysis, among all echocardiographic techniques, 3D PISA showed the highest agreement with CMR [45].

Garg et al. proposed thresholds for CMR severity grading compared with echocardiography as a practical scheme for clinical use [38].

### 4.3. CT

Quantification of MR with CT is feasible but not well established. Two methods exist: the first one is the stroke volume method. A full cycle (0–100%) CT scan is mandatory.

It consists in calculating total stroke volume of the left and right ventricles (end-diastolic volume minus end-systolic volume), with regurgitant volume calculated as the difference between the left and right ventricular stroke volumes, provided that no other valve dysfunction is present [46].

Previous studies reported (Table 3) a good correlation of the regurgitant volume and fraction via CT with cardiac magnetic resonance and/or echocardiography [47].

However, this method is rarely used clinically but could have a role in patients with poor echo windows and if cardiac magnetic resonance imaging is contraindicated.

#### Anatomical Regurgitant Orifice Area (AROA)

Direct planimetry of the anatomic regurgitant orifice area on systolic frames is feasible: MV must be reconstructed into a double oblique plan parallel to the mitral anulus that transects the narrowest mitral regurgitant orifice. Measurements are usually made at 15—25% RR. This method relies on a purely anatomical assessment and does not consider the flow assessment.

First experiences with 16-detector row CT showed that the mitral regurgitant orifice area measured on CT correlated strongly with the transesophageal echocardiography regurgitant jet length and VC [48].

Following studies proved that CT provides strong agreement with qualitative echocardiographic parameters but only a moderate correlation with quantitative echocardiographic parameters of chronic MR [49]. Cardiac computed tomographic angiography slightly overestimated mild MR while slightly underestimating severe MR.

When performed as single-source, 64-slice CT was limited to a temporal resolution of 165 milliseconds, so the point in systole at which the mitral leaflet regurgitant orifice is at its smallest was likely to be missed by CT in many cases. Current technology has overcome this single limitation, but AROA measurements remain technically challenging.

## 5. Anatomical Evaluation: Role of Imaging in Etiology and Mechanism Assessment

### 5.1. TTE

With transthoracic echocardiography, it is possible to obtain a comprehensive scan of the mitral valve anatomy to determine whether the patient has mitral regurgitation, to identify its origin, and to define the etiology of regurgitation and tissue quality, to evaluate the probability of a successful mitral valve repair [50].

For two-dimensional echocardiography, multiple standard views are required to achieve a comprehensive visualization and assessment of the mitral valve. In particular, the four-chamber, intercommissural, two-chamber, and three-chamber views should be obtained. The intercommissural view allows the valve to be assessed in the mediolateral direction, from the medial to the lateral commissure, whereas the three-chamber view provides an anteroposterior assessment of the valve, particularly of the A2 and P2 scallops.

Color-Doppler assessment also helps elucidate the mechanism of mitral regurgitation. In fact, the origin and direction of the regurgitant jets, evaluated across all these views, provide important information about the underlying mechanism. Eccentric jets are generally associated with leaflet prolapse or restriction, whereas centrally directed, vertically oriented jets are more commonly related to annular dilatation or functional mechanisms.

In the parasternal long-axis view, the mitral valve is typically bisected, allowing visualization of the A2–P2 scallops. However, by tilting the transducer toward the tricuspid valve and the RV inflow, the A3–P3 region comes into view. Conversely, tilting the transducer toward the pulmonary valve and the RV outflow allows visualization of A1–P1 [50]. After performing an anatomic assessment of the leaflets without color Doppler, adding color and sweeping the transducer from the RV outflow toward the RV inflow helps identify the exact location of the regurgitant jet. In addition, the parasternal long-axis view allows a measurement of the anteroposterior diameter of the MA in end-systole. In this view, M-mode can be applied to demonstrate the timing and extent of leaflet excursion.

In the parasternal short-axis view, the entire anterior and posterior leaflets are displayed, along with the anterolateral and posteromedial commissures. Laterally, the A1–P1 scallops are seen, A2–P2 are central, and A3–P3 are medial. As in the long-axis assessment, an initial grayscale evaluation should be followed by color Doppler to confirm the presence and location of any regurgitant jet. It is important to consider the saddle-shaped geometry of the MA; jets may be missed in the short-axis view if the imaging plane intersects only the leaflet tips.

Using a three-dimensional probe with X-plane mode allows for a more accurate biplane assessment from the short-axis view. Positioning the X-plane cursor at the center of the valve generates a derived parasternal long-axis view that displays A2–P2. Shifting the X-plane line toward the medial commissure reveals A3–P3, and moving it toward the lateral commissure reveals A1–P1.

From the short-axis view, it is also important to assess the papillary muscles, which may be hypertrophic or bifid, reduced in number as in a parachute mitral valve, or, conversely, supernumerary. In the apical four-chamber view, the anterior leaflet is visualized closer to the septum and the posterior leaflet adjacent to the inferolateral wall. The scallops typically imaged in this view are A3/A2 and P1. The apical four-chamber view also allows an assessment for leaflet tethering and, when present, enables a measurement of the tenting area [50].

In the apical two-chamber view, the mitral valve leaflets visualized are P3 adjacent to the inferior wall, P1 adjacent to the anterior wall, and anatomically close to the left atrial appendage, with A2 seen in the middle of the valve. With 3D TTE using X-plane, placing the reference line at the center of the valve in the apical two-chamber view generates a derived four-chamber view that displays the A2–P2 scallops. Shifting the X-plane line toward the inferior wall provides visualization of A3–P3, while moving it laterally toward the left atrial appendage yields the A1–P1 scallops [50].

In the apical three-chamber view, which is similar to the parasternal long-axis view, the A2–P2 scallops are visualized. By performing a comprehensive evaluation of the mitral valve, it is possible to describe leaflet motion—whether normal, excessive, or restricted. Leaflet thickness and calcification should also be assessed: leaflet thickness should be measured and reported, and the extent and distribution of calcification should be described.

TTE also plays a key role in assessing the consequences of mitral regurgitation. Specifically, a biplane measurement of the LA volume is recommended. In acute mitral regurgitation, the rapid onset may result in a normal or only mildly enlarged LA, whereas in chronic mitral regurgitation, the LA is often severely dilated. Severe chronic primary mitral regurgitation increases the LV preload, leading, over time, to chamber dilation, eccentric hypertrophy, and eventually impaired LV function. A biplane Simpson assessment of ventricular volumes is recommended, as well as indexing these volumes to the patient’s body surface area (BSA). Furthermore, increases in LA pressure secondary to severe mitral regurgitation are reflected by elevated pulmonary artery capillary wedge pressure, which in turn leads to increased systolic pulmonary artery pressure (SPAP).

### 5.2. TEE

A comprehensive TEE examination includes mid-esophageal and, when needed, transgastric views, both with and without color Doppler.

During the TEE examination of the mitral valve, it is of paramount importance to acquire a complete set of images, following a specified protocol. Using off-axis imaging will cause the loss of the known spatial relationships.

As in transthoracic echocardiography, the use of multiple standard views in transesophageal echocardiography allows an accurate orientation within the anatomy of the mitral valve and facilitates an assessment of the mechanisms of regurgitation. By combining anatomical evaluation with the analysis of the origin and direction of the regurgitant jets, it is possible to characterize mitral valve anatomy, identify the underlying lesions, and determine the mechanism of regurgitation.

In transesophageal echocardiography, both two-dimensional and three-dimensional imaging techniques should be used to achieve a comprehensive assessment of mitral valve anatomy. With two-dimensional imaging, multiple standard views are required to fully evaluate the valve. The four principal views are the mid-esophageal four-chamber, intercommissural, two-chamber, and three-chamber views.

In the mid-esophageal (ME) five-chamber view, leaflet segments A2 and P2–1 can be evaluated.

In the ME four-chamber view, the probe is retroflexed to visualize the true LV apex; the LVOT is excluded from the sector, and the image is centered on the mitral valve. This view represents an oblique imaging plane through the valve and typically displays A2–1 and P1 but it can vary with probe depth.

A systematic sweep of the mitral valve can then be performed by gradually advancing the probe: first to visualize A2–P2 and further to visualize A3–P3, corresponding to high, mid, and low esophageal levels. It is important to note that these are oblique planes through the valve rather than views perpendicular to the coaptation line. They are useful for obtaining an overview of lesions and their approximate location; confirmation requires rotation of the multiplane angle.

In the ME commissural view (≈60°), the segments P3, A2, and P1 are visualized; the left atrial appendage lies adjacent to P1, while P3 is adjacent to the interatrial septum.

In the ME two-chamber view (≈90°), P3 and all three anterior leaflet segments (A3–A2–A1) are seen.

In the ME three-chamber view (≈130°), A2 (adjacent to the aortic valve) and P2 are visualized.

From the transgastric 0° short-axis view, all six mitral scallops and both commissures can be observed en-face from the LV perspective. By increasing the imaging angle toward approximately 90°, the transgastric long-axis view is obtained, allowing a detailed assessment of the subvalvular mitral apparatus.

One of the most important technological innovations in the evaluation of the mitral valve in echocardiography is the introduction of real-time three-dimensional imaging. Real-time three-dimensional echocardiography (RT3DE) provides enhanced visualization of mitral valve anatomy and function. Among the available acquisition modes, 3D zoom is commonly used to obtain an en-face view of the valve from either the atrial or ventricular perspective. The relatively small acquisition volume allows higher frame rates and may permit single-beat acquisition.

The most used display modality is the surgical view that is represented with the aortic valve at 12 o’clock.

In the commissural view at approximately 60°, using the X-plane mode, it is possible to rapidly assess lesions at A3–P3 when the plane intersects the medial region, A2–P2 in the central portion, or A1–P1 laterally. This approach allows for quick and precise localization of prolapse or flail segments, facilitating efficient diagnostic workflow and pre-procedural planning [50].

### 5.3. CMR

Cardiovascular magnetic resonance (CMR) provides a comprehensive and complementary assessment of the mitral valve (MV), subvalvular apparatus, and myocardial consequences of mitral valve disease. Dedicated long-axis cine imaging enables a detailed evaluation of leaflet morphology and motion, including prolapse, flail, thickening, perforation, clefts, and masses, as well as abnormalities of the subvalvular apparatus, such as chordal rupture and papillary muscle abnormalities. CMR can therefore contribute to defining the mechanism of mitral regurgitation (MR) and to distinguishing primary from secondary etiologies. Although leaflet calcification may appear as areas of signal loss, CMR is less sensitive than computed tomography for its characterization.

Dedicated long-axis and en-face cine views can identify leaflet prolapse or flail and may help localize the involved leaflet or scallop. Nevertheless, the spatial resolution of conventional cine CMR, typically acquired with a slice thickness of approximately 5–6 mm, remains lower than that of echocardiography and may limit accurate scallop-level differentiation between segmental prolapse and flail leaflet [51]. This limitation should be considered particularly when a detailed anatomical characterization is required for procedural planning.

Beyond valve morphology, CMR is the reference standard for the non-invasive quantification of cardiac chamber volumes and ventricular function, providing highly reproducible assessment of the hemodynamic consequences of MR and enabling a longitudinal evaluation of cardiac remodeling after mitral valve intervention [52]. Reductions in left ventricular dimensions following mitral valve correction have been demonstrated after both surgical and transcatheter intervention, while the CMR-derived mitral regurgitant volume has been shown to predict postoperative left ventricular end-diastolic volume and the magnitude of reverse remodeling [53].

CMR also provides a robust assessment of mitral annular disjunction (MAD), particularly when echocardiographic findings are equivocal, and allows an evaluation of both its longitudinal extent and circumferential distribution. MAD is commonly measured on end-systolic long-axis cine images as the separation between the left atrial wall–mitral leaflet hinge point and the adjacent basal left ventricular myocardium [54]. A threshold of ≥1 mm has been used in several CMR studies; however, small degrees of disjunction are relatively common, and their clinical significance appears to depend on the extent and location of MAD, as well as the presence of additional phenotypic abnormalities. MAD is increasingly recognized as one component of the arrhythmic MVP phenotype, although its independent contribution to ventricular arrhythmogenesis remains incompletely defined [55]. A representative three-chamber cine view showing bileaflet MVP and an end-systolic MAD measurement is provided in Figure 6.

An additional major strength of CMR is myocardial tissue characterization. Late gadolinium enhancement (LGE) enables the detection and quantification of focal replacement fibrosis, providing information that is not available from conventional echocardiography. In patients with mitral valve prolapse (MVP), replacement fibrosis most frequently involves the papillary muscles and the basal inferior or inferolateral left ventricular myocardium and has been associated with ventricular arrhythmias and adverse cardiovascular outcomes [22,56]. A representative LGE example of mid-wall fibrosis in the inferolateral myocardium is shown in Figure 7.

Myocardial tissue characterization is also particularly relevant in secondary, and especially ischemic, MR. LGE-CMR allows the quantification of the extent and distribution of myocardial infarction and scars involving the left ventricle and papillary muscles, thereby providing additional prognostic information beyond MR severity alone. In ischemic cardiomyopathy, the myocardial infarct size and MR severity have been shown to interact in determining clinical risk, with a large scar burden associated with adverse outcomes across the spectrum of ischemic MR severity [57]. Post-contrast long-axis LGE images demonstrating non-ischemic septal fibrosis and the absence of ischemic scar are shown in Figure 8.

### 5.4. CT

Cardiac CT is capable of providing detailed anatomical information on the entire valvular apparatus (annulus, leaflets, and subvalvular apparatus).

To accurately assess the MV anatomy and geometry using CT, the reconstructed short-axis view should be generated after precisely selecting the MA plane in four- and two-chamber views. Koo et al. introduced the “en face” view of the MV, i.e., the surgeon’s view. By setting the images on the “en face” view, either perpendicular or parallel to the coaptation line, the MV sagittal or coronal views, respectively, can be obtained by using a multiplanar reformatting display technique [58].

The MA has a complex, saddle-shaped three-dimensional geometry. Its dimensions are dynamic and therefore should be measured in both systolic and diastolic phases.

It consists of an anterior horn that is continuous with the aortic valve (aorto-mitral continuity) and a posterior horn that is formed by the insertion of the posterior mitral leaflet at the atrio-ventricular junction. The nadirs of the saddle are located near the fibrous trigones, which are defined as the regions of collagenous thickening at the ends of the aorto-mitral curtain. The medial fibrous trigone is continuous with the membranous interventricular septum. It is important to note that the anterior horn of the MA encroaches on the LVOT, and therefore, inclusion of this component in sizing of the annulus will lead to device encroachment into the LVOT and potentially fatal LVOT obstruction after transcatheter mitral valve replacement.

To avoid LVOT obstruction, a D-shaped annulus can be assumed with the medial and lateral fibrous trigones connected by a virtual straight line (Figure 9) [59].

In patients with MR, annular dimensions are significantly larger, particularly in those with MVP, and annular geometry is distorted with septal–lateral expansion. Similar to previous studies using conventional methods for quantifying the annular size, the MA area was 18% larger in patients with MVP compared with vFMR [60].

CT provides a 3D data set with outstanding spatial resolution that allows good-to- excellent visualization of the anatomic details of the mitral leaflets with good agreement with echocardiography and surgical findings in the diagnosis of mitral valve abnormalities (Figure 10).

Anterior and posterior leaflets with their scaffold are identifiable via CT and seen in both reconstructed short-axis and long-axis views.

In primary MR, CT allows the identification of prolapse, flail, calcification, endocarditic lesions, and congenital mitral valve abnormalities.

Specifically, CT can reliably detect the MVP that is best visualized in systolic phases. Feuchtner et al. demonstrated that CT can characterize MVP with sensitivity, specificity, positive predictive, and negative predictive values of 96%, 93%, 93%, and 96%, respectively, when compared with echocardiography [61].

Koo et al. reported that the best quality images were obtained during the phases at 25% to 35% of the cardiac cycles in patients with MV prolapse. Two- and three-chamber views can be used to identify leaflet thickening (>5 mm) and a flail leaflet, both seen in the context of mitral prolapse (Figure 11) [58]. Mitroannular disjunction (MAD), frequently associated with myxomatous bi-leaflet prolapse (Barlow disease), can also be identified during systole.

Cardiac CT allows an accurate assessment of the extent and distribution of mitral annular calcification (MAC), which may also be quantified using an Agatston-type calcium scoring approach similar to that applied for coronary artery calcification [62]. However, in the presence of extensive MAC, identification of the interface between the mitral annulus and the blood pool may become more challenging, potentially affecting the accuracy of annular measurements. MAC also increases the technical complexity of both surgical and transcatheter interventions, with a higher risk of atrioventricular groove disruption, paravalvular leak, and left ventricular outflow tract (LVOT) obstruction. In addition to a calcium burden assessment, Guerrero et al. proposed a CT-based MAC score (Figure 12) incorporating four anatomical features—average calcium thickness, circumferential extent, involvement of the fibrous trigones, and leaflet calcification—to provide a standardized classification of MAC severity. The score ranges from 0 to 10 and categorizes MAC as mild (≤3 points), moderate (4–6 points), or severe (≥7 points), thereby offering a reproducible framework for anatomical characterization and procedural planning, particularly in patients considered for transcatheter mitral valve interventions [63,64].

Also, in rheumatic disease, the mitral valve calcium score (MVCS) proved to be an independent predictor of both the immediate procedural success of percutaneous mitral commissurotomy and long-term survival free of mitral reintervention [65].

In the context of infective endocarditis, cardiac CT can accurately detect vegetations, abscesses or aneurysms, leaflet perforation, and valvular dehiscence.

Computed tomography has proven to be able to detect vegetations with a maximum length as small as 6 mm. This capability is clinically relevant. A vegetation size ≥10 mm is associated with an increased embolic risk; however, surgical decision-making should integrate the vegetation size with other clinical factors, including previous embolic events, valve dysfunction, infection control, and the overall clinical context [66].

Kotaro et al. found that CT and TTE had comparable ability to detect vegetations, although CT had lower sensitivity than TEE. Moreover, in evaluating the capability of CT to depict vegetations and their locations in the aortic and mitral valves, sensitivity and PPV for vegetations in the aortic valve were observed to be 100%, with lower sensitivity for detecting vegetations on the mitral valve [67]. One possible explanation is that the presence of the chordae tendineae and papillary muscles associated with the mitral valve can, in some cases, complicate the distinction between native muscular structures and vegetative lesions. In contrast, the identification of vegetations on the aortic valve may be more straightforward, as the aortic valve lacks comparable anatomical structures that could interfere with lesion recognition.

In addition, CT seems to have an advantage over TEE in assessing patients with more extensive calcification of the valves with perivalvular extensions such as abscess and pseudoaneurysm, or in those with IE involving prosthetic valves, in which acoustic shadowing can decrease the sensitivity of TEE [68]. CT also provides supplementary information related to extra-cardiac foci of infection.

Finally, cardiac CT imaging might be performed to better define rare congenital abnormalities seen at initial echocardiography such as mitral clefts (isolated or associated with endocardial cushion defects), parachute mitral valve, and double-orifice mitral valve.

In secondary MR, CT allows a full geometric assessment of the valve, including the leaflet length, area, tenting height, and coaptation angle.

These measurements are important for understanding the mechanism of MR and guiding intervention. Furthermore, multiphasic ECG-gated cine cardiac CTA demonstrated that thinned LV segments were associated with less pronounced reverse remodeling at 1 month after the procedure, whereas higher baseline LVEDVi and RVEDVi were associated with greater reductions in LVEDVi and RVEDVi, respectively [69].

## 6. Practical Role of Multimodality Imaging in Mitral Regurgitation

TTE remains the first-line examination for defining the etiology, mechanism, and severity of MR. However, in contemporary clinical practice, multimodality imaging has become indispensable for a comprehensive assessment of mitral regurgitation. TTE, CMR, and CT provide complementary information on the anatomy, mechanism, and severity of MR, allowing for a more accurate and integrated characterization of the disease and ultimately guiding the selection of the most appropriate treatment strategy for each patient (Figure 13).

TEE is primarily used when a more detailed assessment of leaflet and scallop anatomy, repairability, endocarditis, or procedural planning is required. CMR should be considered a targeted second-level examination, rather than a routine test, when echocardiographic findings are inadequate or discordant with symptoms or cardiac chamber remodeling [14,37,38,70].

A transition from TTE to CMR is particularly useful in four clinical scenarios: (i) a suboptimal acoustic window, incomplete quantitative data, or internal discordance among echocardiographic parameters [14,37,38,70]; (ii) complex regurgitant physiology, including multiple, eccentric or non-holosystolic jets and a non-circular or dynamic regurgitant orifice, in which the assumptions underlying the proximal isovelocity surface area (PISA) method may be unreliable [38,42,70,71]; (iii) associated cardiomyopathy or otherwise unexplained ventricular dysfunction, when accurate biventricular volumes, systolic function, and myocardial tissue characterization are required [38,40,41]; and (iv) a suspected arrhythmic MVP phenotype, particularly in the presence of complex ventricular arrhythmias, unexplained syncope, or high-risk morphological features, when CMR can identify papillary muscle or inferolateral myocardial fibrosis [22,38,72].

The two modalities quantify MR using different principles. Echocardiographic PISA-derived EROA is an instantaneous measurement, while the regurgitant volume (RVol) is derived from geometric and Doppler assumptions. These measurements may therefore be affected by systolic variation, eccentricity, multiple jets, and non-hemispheric flow convergence. The standard indirect CMR method calculates RVol as the left ventricular stroke volume minus aortic forward flow measured via phase-contrast imaging and RF as RVol divided by left ventricular stroke volume. CMR thus measures the total regurgitant burden per cardiac cycle and is independent of jet direction or number. However, arrhythmia, phase-offset errors, concomitant aortic regurgitation, and intracardiac shunts require adapted acquisition or alternative quantification strategies [38,70,71].

CMR-derived thresholds should not be regarded as direct substitutes for guideline-based echocardiographic criteria. In primary MR, Myerson et al. showed that an RVol > 55 mL and an RF > 40% were associated with progression to symptoms or another indication for surgery [39]. Penicka et al. confirmed the prognostic value of the CMR-derived MR burden and used an RVol ≥ 60 mL to define severe organic MR [19]. Accordingly, the commonly proposed CMR severe range for primary MR is an RVol > 55–60 mL and an RF > 40% [19,38,39]. In secondary MR, Wang et al. identified outcome-based RF thresholds of ≥20% and ≥35% for moderate and severe risk categories, respectively [40], whereas Tayal et al. showed that the prognostic significance of an RF ≥ 30% was modified by the myocardial scar burden [41]. Therefore, CMR cut-offs remain proposed or outcome-informed values and should be interpreted within an integrated assessment of MR mechanism, ventricular remodeling, and myocardial tissue characteristics.

Computed tomography (CT) provides complementary information when a detailed anatomical definition is required. In patients with mitral annular calcification (MAC), CT accurately depicts the location, circumferential extent, thickness, and distribution of calcium, as well as its involvement of the annulus, leaflets, and adjacent structures, thereby supporting an assessment of surgical complexity and procedural feasibility [63,73]. CT is particularly important for the pre-procedural planning of transcatheter mitral valve replacement and valve-in-MAC procedures because it enables annular and landing-zone measurements, device sizing, assessments of the spatial relationship with the left ventricular outflow tract, and virtual valve simulation, including prediction of the neo-left ventricular outflow tract (LVOT) and the risk of post-procedural LVOT obstruction [63,73,74]. For transcatheter edge-to-edge repair, three-dimensional TEE remains the principal modality for leaflet assessment and intraprocedural guidance, whereas CT may be considered selectively when extensive calcification or complex annular anatomy requires further definition [63,73]. Accordingly, CT should be regarded primarily as an anatomical and procedural-planning tool, rather than as a routine alternative to echocardiography or CMR for MR quantification.

## 7. Conclusions

Mitral regurgitation is a complex and heterogeneous valvular disease in which accurate assessment requires the integration of valve anatomy, regurgitation severity, ventricular and atrial remodeling, and the underlying myocardial substrate. Transthoracic echocardiography remains the cornerstone and first-line imaging modality, while TEE, CMR, and cardiac CT should be regarded as complementary, question-driven tools rather than competing techniques. TEE provides superior anatomical definition and is essential when detailed characterization of the mitral apparatus or planning of surgical and percutaneous interventions is required. CMR offers the robust and reproducible quantification of regurgitant burden and cardiac remodeling, providing additional value through myocardial tissue characterization, particularly when echocardiographic findings are inconclusive or discordant. Cardiac CT adds high-resolution anatomical information, playing an important role in the assessment of annular calcification and in planning increasingly complex transcatheter procedures. Overall, a tailored multimodality approach, in which each technique is selected according to the specific clinical question, may improve diagnostic confidence, refine risk stratification, and support timely Heart Team decision-making and individualized treatment. Further standardization and prospective validation of advanced imaging parameters will be important to define their optimal integration into future MR management pathways.

## Figures and Tables

**Figure 1:**
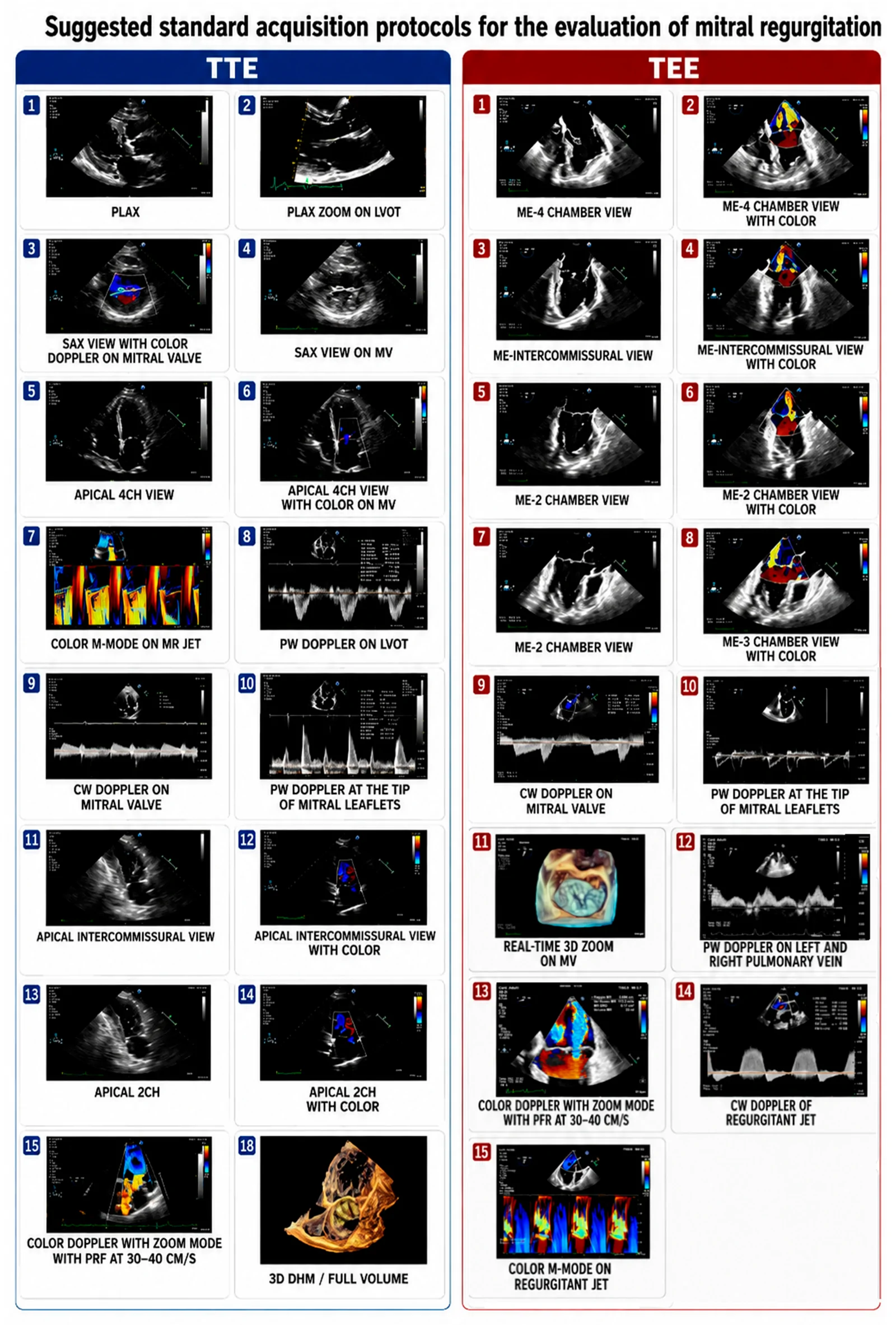
Acquisition protocol for echocardiographic assessment of mitral regurgitation: minimal TTE and TEE acquisition protocol. The figure illustrates a practical workflow for the echocardiographic assessment of MR, starting with TTE as the first-line imaging modality and proceeding to TEE when further anatomical or diagnostic assessment is required. TTE provides an initial evaluation of mitral valve morphology, the MR mechanism and severity, quantitative parameters, and left atrial and ventricular remodeling. TEE allows a more detailed assessment of valve anatomy and regurgitant jets through systematic 2D and color Doppler imaging, complemented by three-dimensional (3D) imaging and spectral Doppler when appropriate. The overall findings are integrated to define the mechanism and severity of MR and to support subsequent clinical and procedural decision-making. TTE: transthoracic echocardiography; TEE: transesophageal echocardiography; MR: mitral regurgitation.

**Figure 2:**
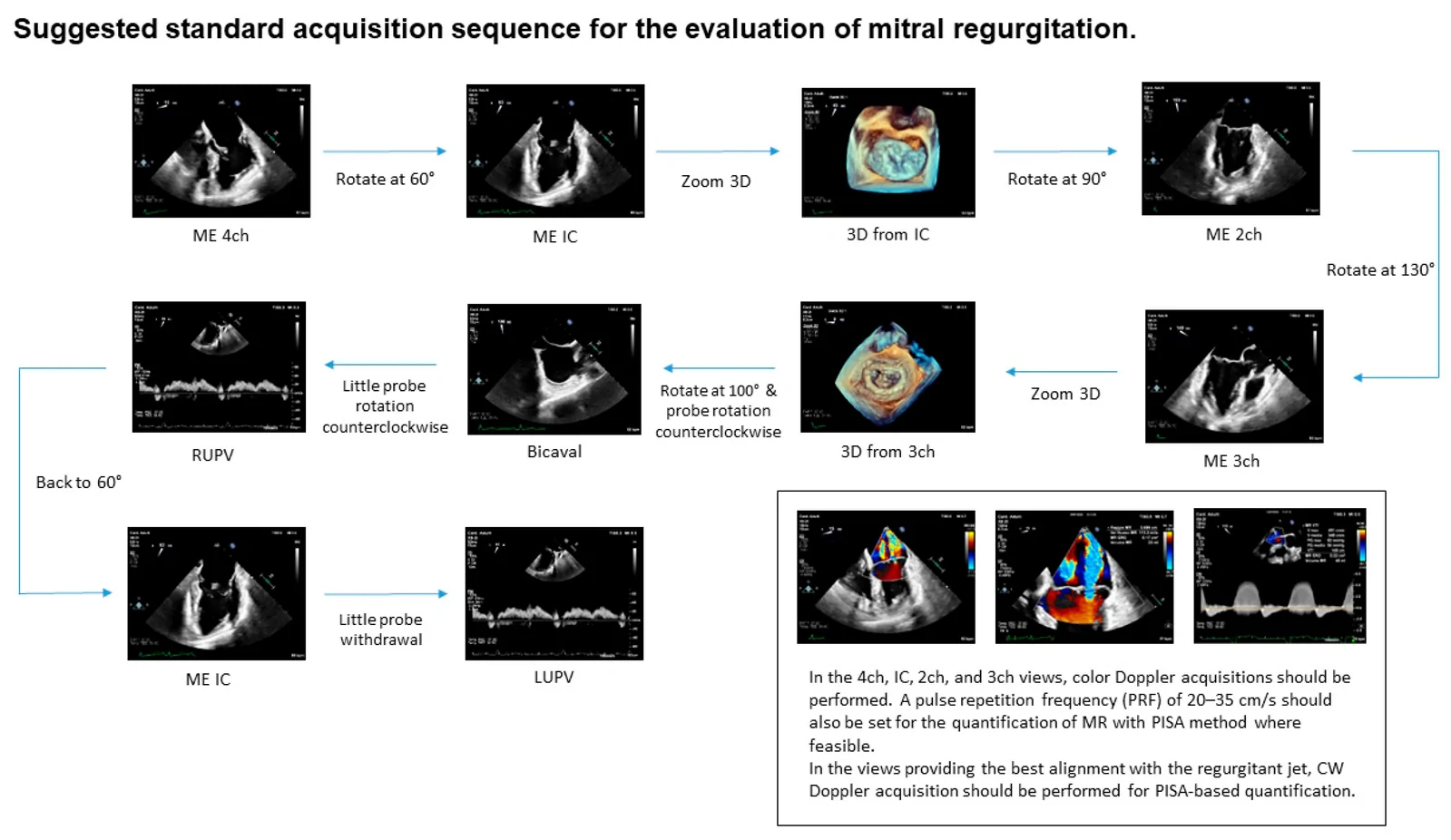
Suggested standard minimal transesophageal echocardiography acquisition sequence for the comprehensive assessment of mitral regurgitation.

**Figure 3:**
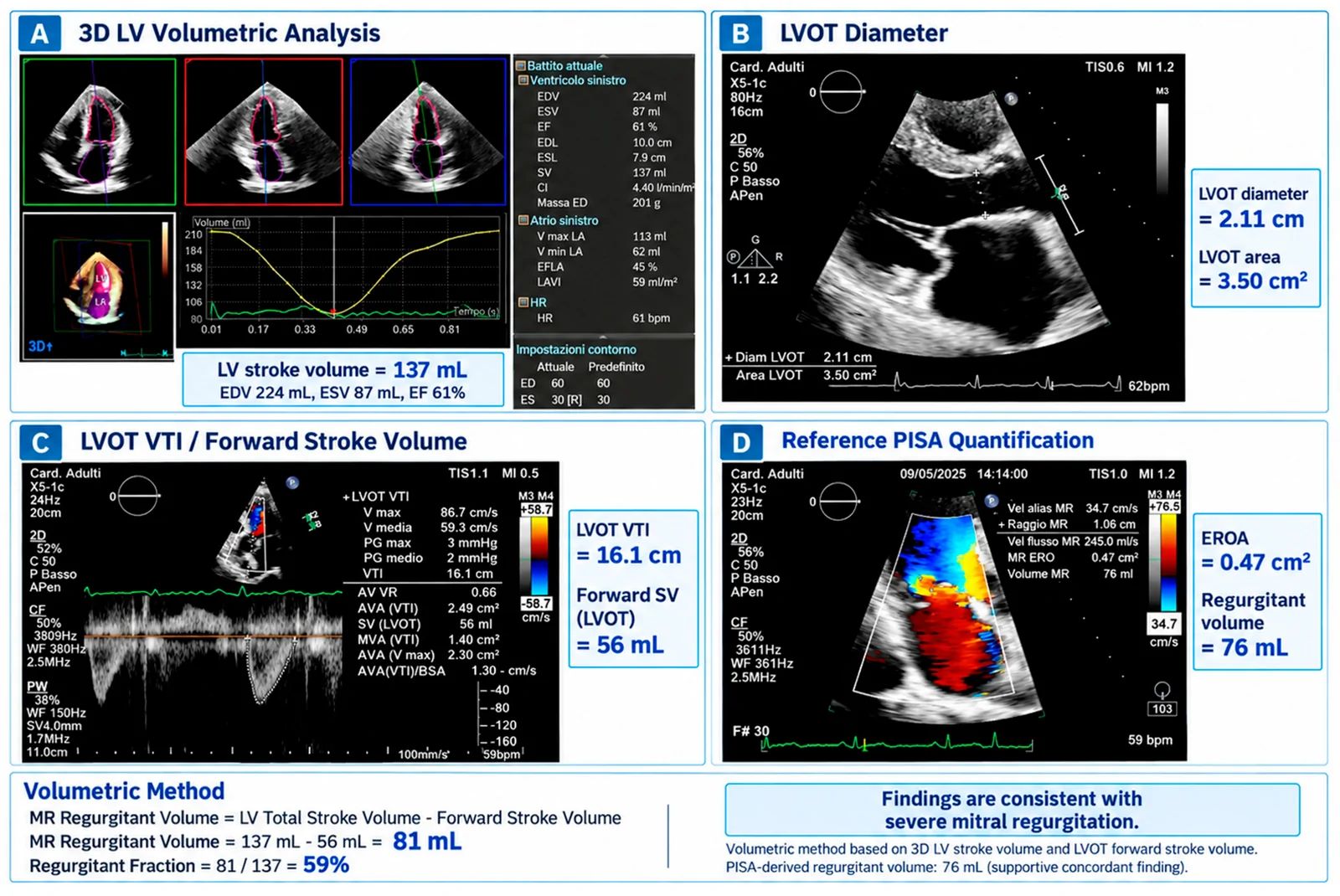
Volumetric quantification of mitral regurgitation. Three-dimensional LV analysis yielded a total stroke volume of 137 mL (EDV 224 mL; ESV 87 mL) (**A**). LVOT diameter was 2.11 cm, corresponding to an area of 3.50 cm^2^ (**B**). LVOT VTI was 16.1 cm, resulting in a forward stroke volume of 56 mL (**C**). Accordingly, volumetric regurgitant volume was 81 mL, and the regurgitant fraction was 59%. PISA analysis showed an EROA of 0.47 cm^2^ and a regurgitant volume of 76 mL (**D**), providing concordant quantitative assessment.

**Figure 4:**
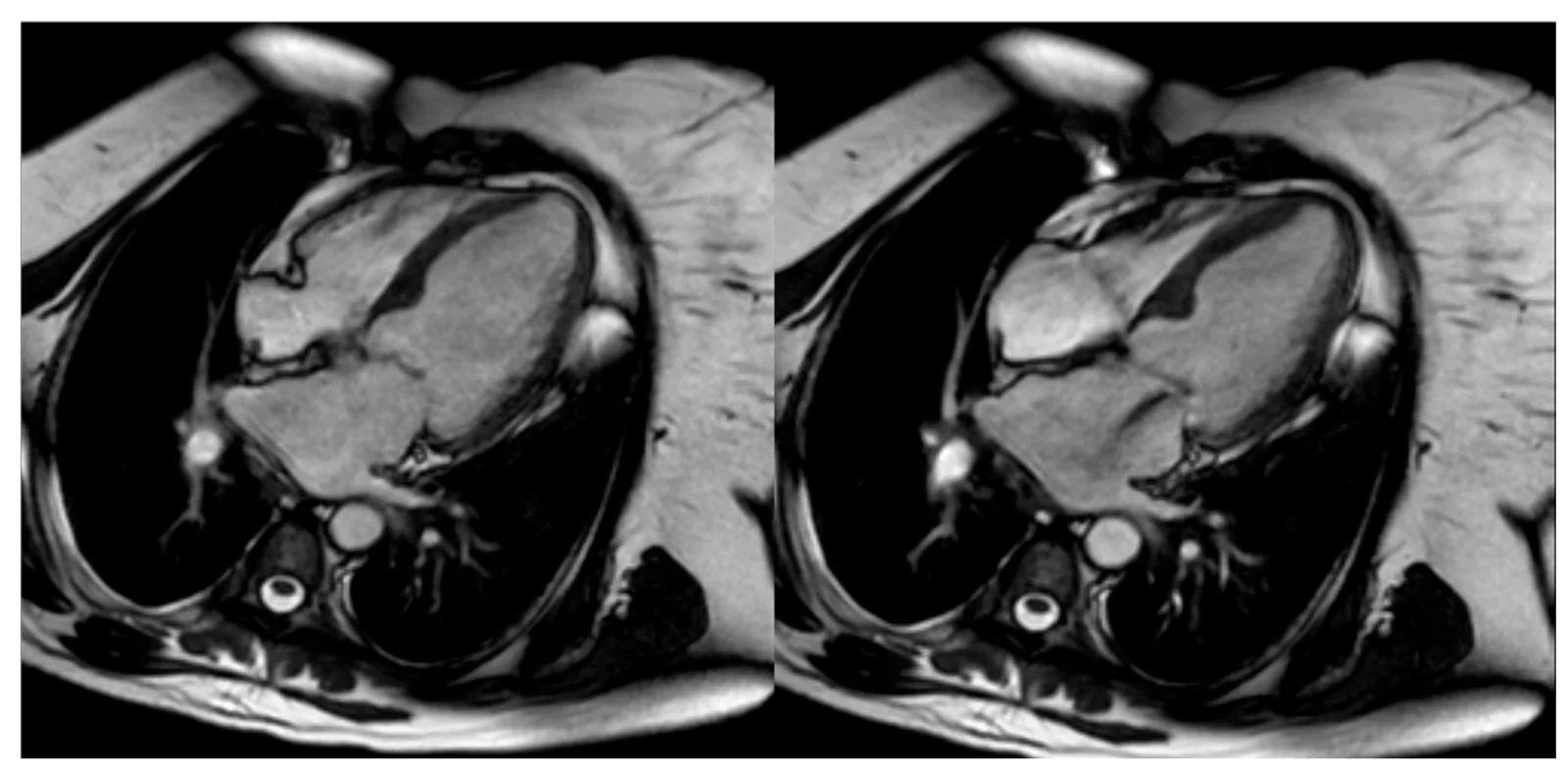
End-diastolic (**right** panel) and end-systolic cine SSFP image (**left** panel) of secondary MR in ischemic cardiomyopathy, three-vessel disease, and severe LV dysfunction (EF 31%).

**Figure 5:**
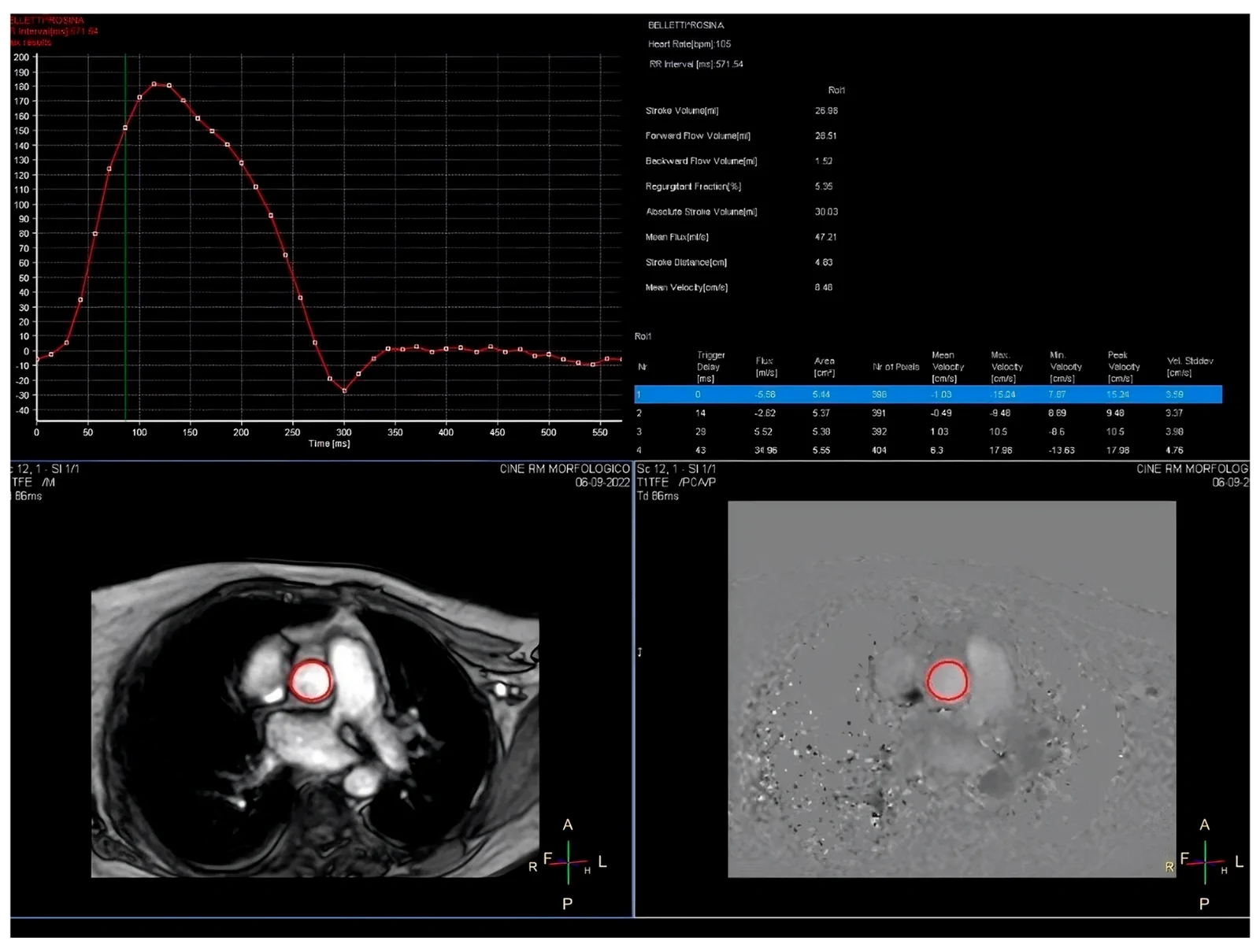
An RVol of 29 mL/beat is calculated subtracting the aortic forward flow volume (measured based on PC sequences) from the LVSV (derived from SSFP short-axis cine images). The resulting RF was 52%, corresponding to severe MR according to the RF criterion.

**Figure 6:**
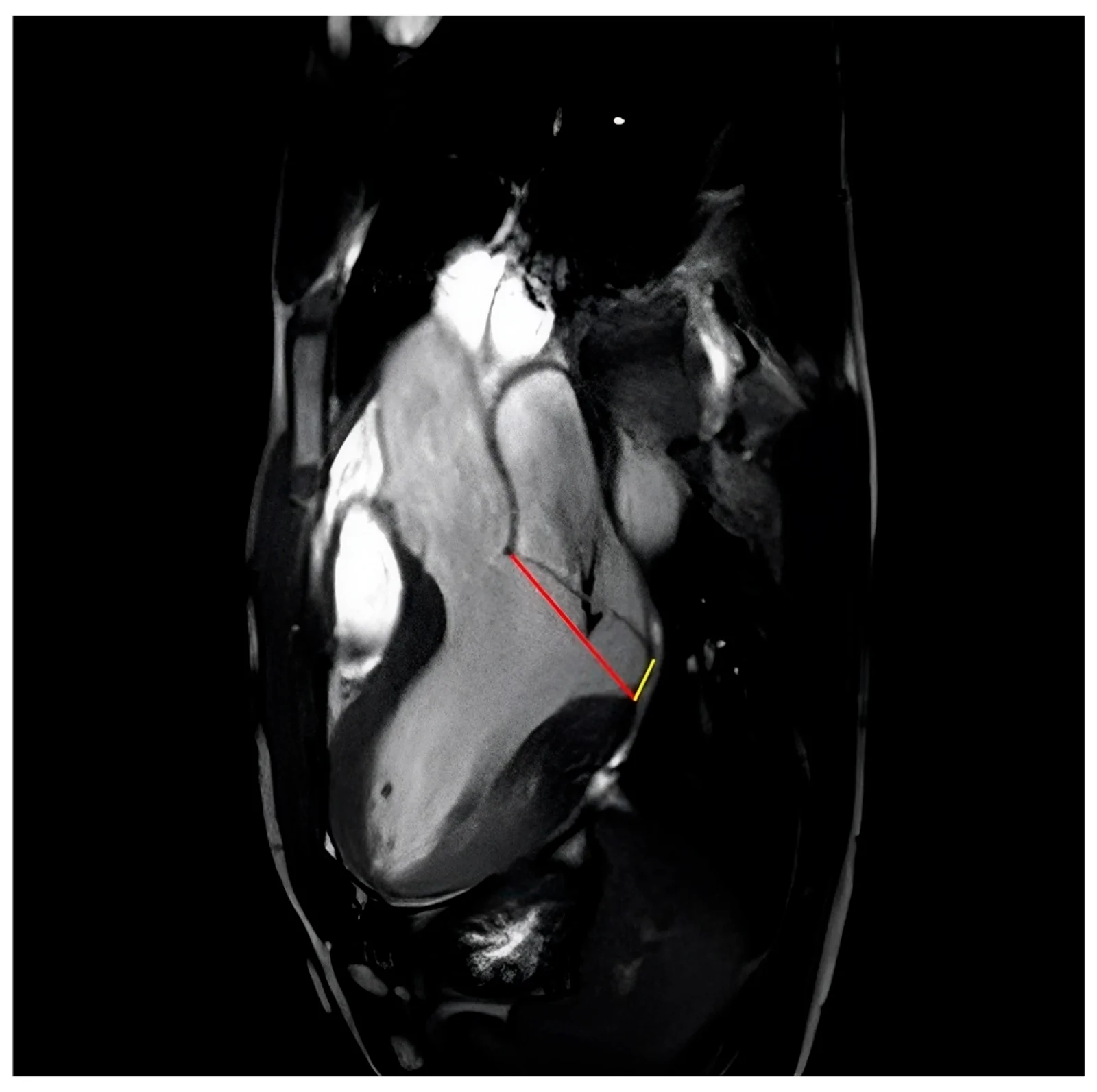
Moderate primary MR in bileaflet MVP (arrhythmic phenotype), visualized in three-chamber view. Prolapse is measured from the annular plane (red line) to the mitral leaflet. Mitral annular disjunction is measured at end-systole from the left atrial wall-posterior leaflet junction to the top of the LV inferobasal wall (yellow line).

**Figure 7:**
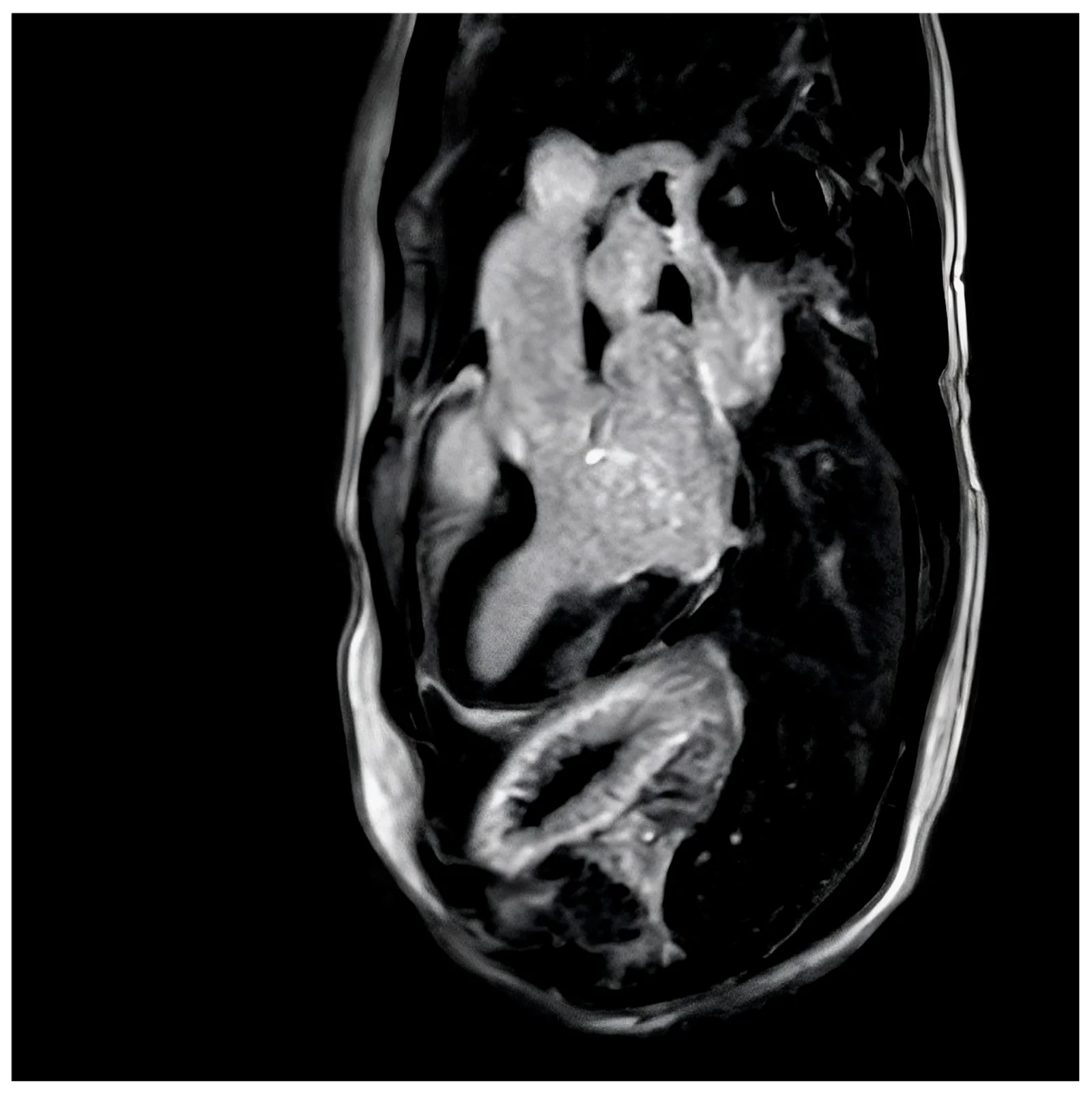
Post-contrast LGE sequence show mid-wall fibrosis of mid inferolateral wall.

**Figure 8:**
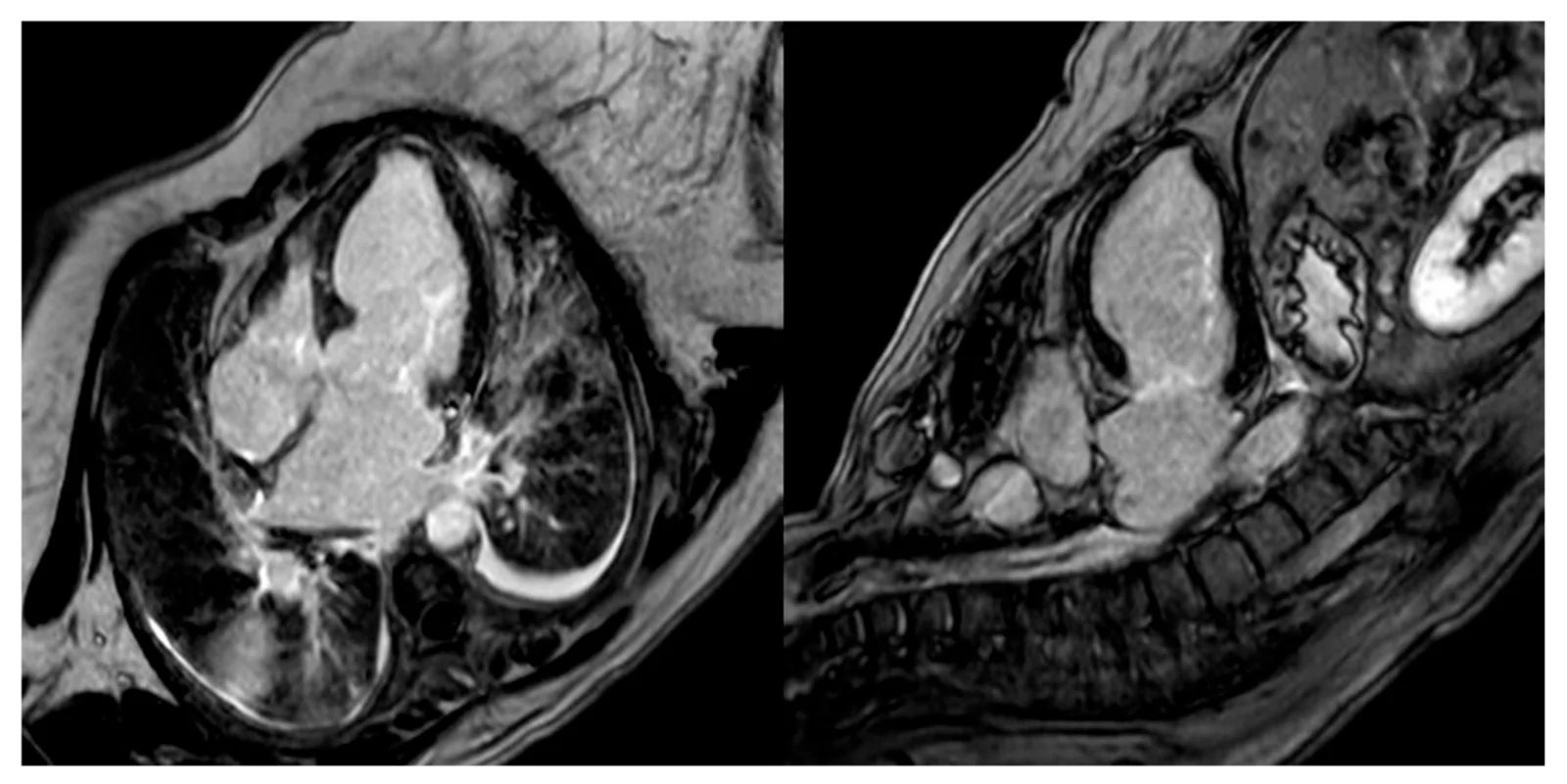
Post-contrast four-chamber (**left** panel) and two-chamber (**right** panel) LGE sequences show areas of non-ischemic mid-wall fibrosis in the septum and absence of ischemic scars.

**Figure 9:**
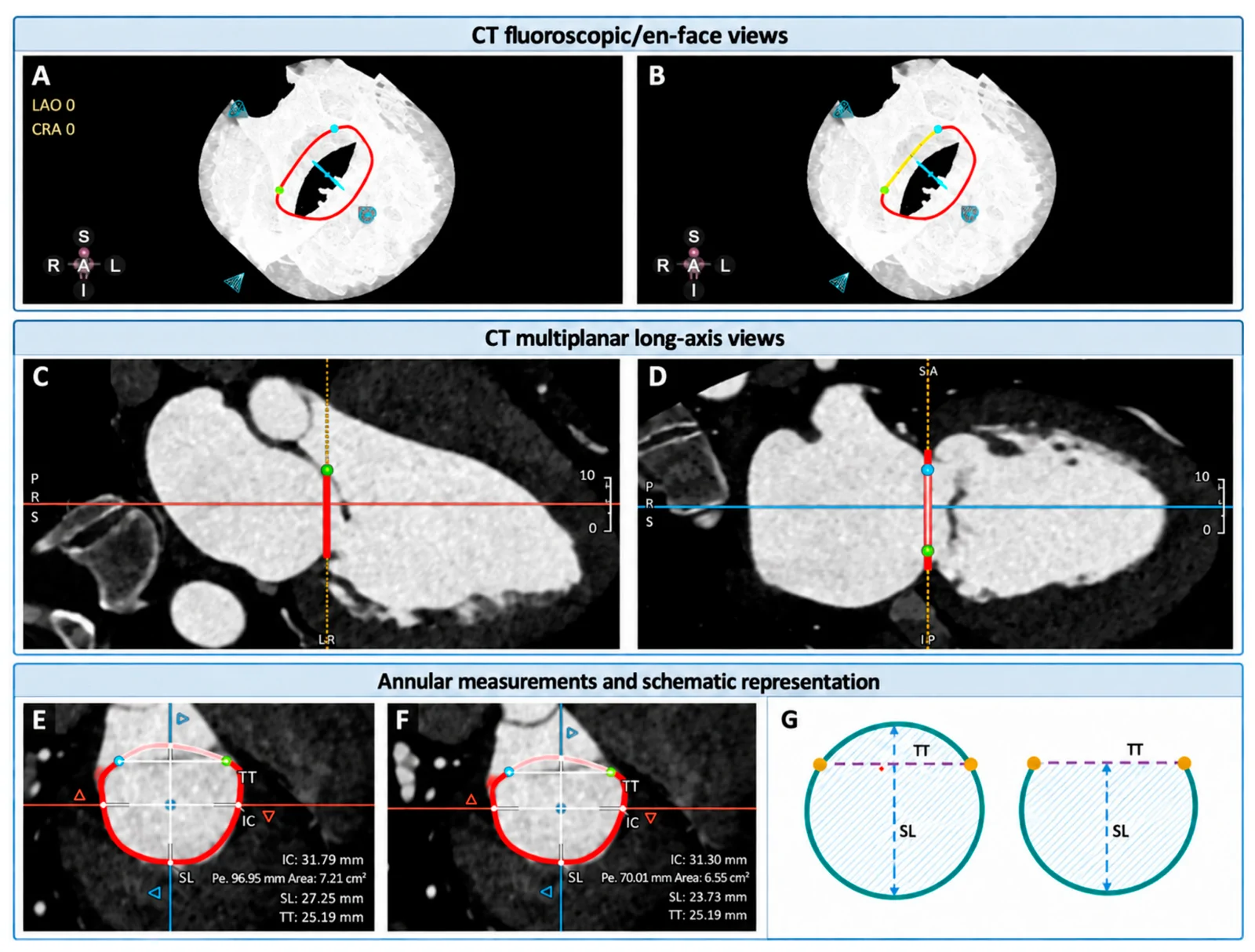
CT-based assessment of mitral annular geometry and measurements. (**A**) Three-dimensional CT reconstruction of the native saddle-shaped mitral annulus displayed in a fluoroscopic/en-face projection, with the annular contour and anatomical landmarks used for subsequent measurements. (**B**) Corresponding D-shaped annular reconstruction obtained by connecting the fibrous trigones and excluding the anterior horn of the saddle-shaped annulus, illustrating the simplified annular geometry used for device sizing. (**C**) Multiplanar CT reconstruction in a three-chamber long-axis view used to identify the mitral annular plane and define the septal–lateral dimension. (**D**) Orthogonal two-chamber long-axis reconstruction used to refine annular alignment and confirm the position of the annular landmarks. (**E**) En-face CT view of the saddle-shaped mitral annulus with manual contouring and measurement of the annular dimensions, perimeter, and area. (**F**) Corresponding D-shaped annular reconstruction, with measurements recalculated after exclusion of the anterior portion of the saddle-shaped contour. (**G**) Schematic representation of the saddle-shaped annulus (**left**) and D-shaped annulus (**right**), highlighting the main dimensions used for CT assessment. TT, trigone-to-trigone distance; SL, septal–lateral diameter; IC, intercommissural diameter.

**Figure 10:**
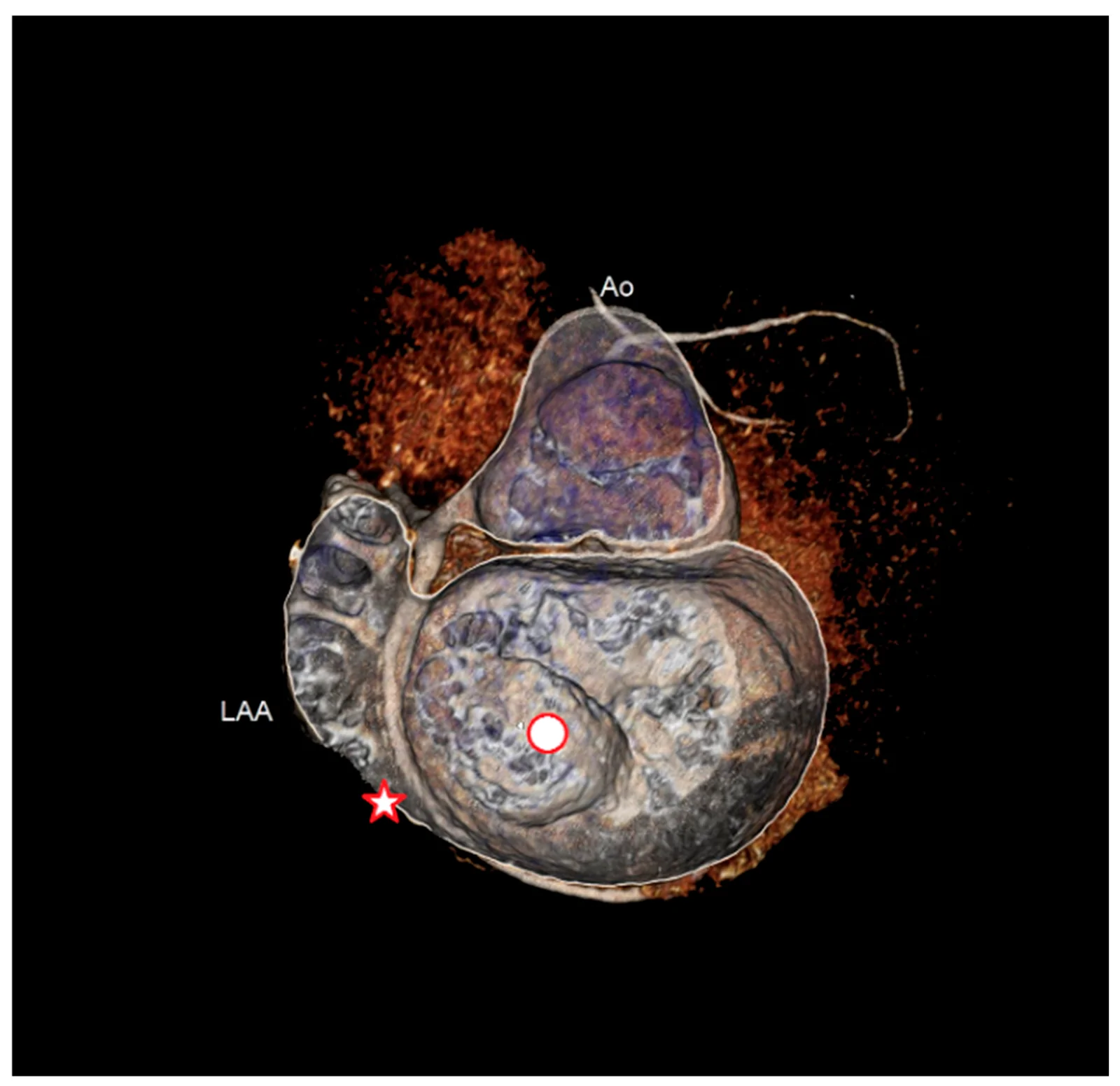
Three-dimensional reconstruction of the mitral valve (late systolic-phase). In this image, the valve is displayed in the classic “surgical” view, as seen from the left atrium. The aortic valve serves as the anterior landmark, while the left atrial appendage serves as the lateral landmark. The dot symbol indicates a large prolapse of the posterior mitral leaflet (P2 scallop). The star symbol indicates the circumflex artery, which runs within the atrioventricular groove and is in close anatomical relationship with the posterior attachment of the mitral valve. LAA: left atrial appendage. Ao: aortic valve.

**Figure 11:**
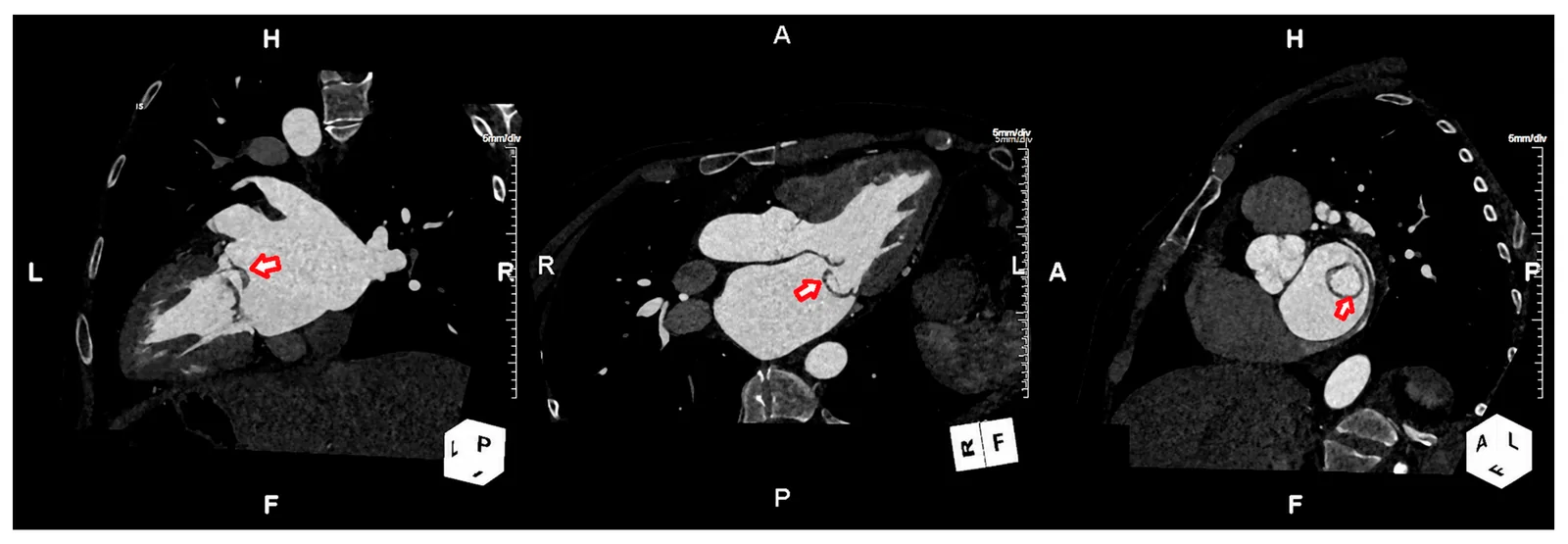
The arrow indicates a flail posterior mitral leaflet in the setting of mitral valve prolapse, shown in two-chamber, three-chamber, and short-axis CT views.

**Figure 12:**
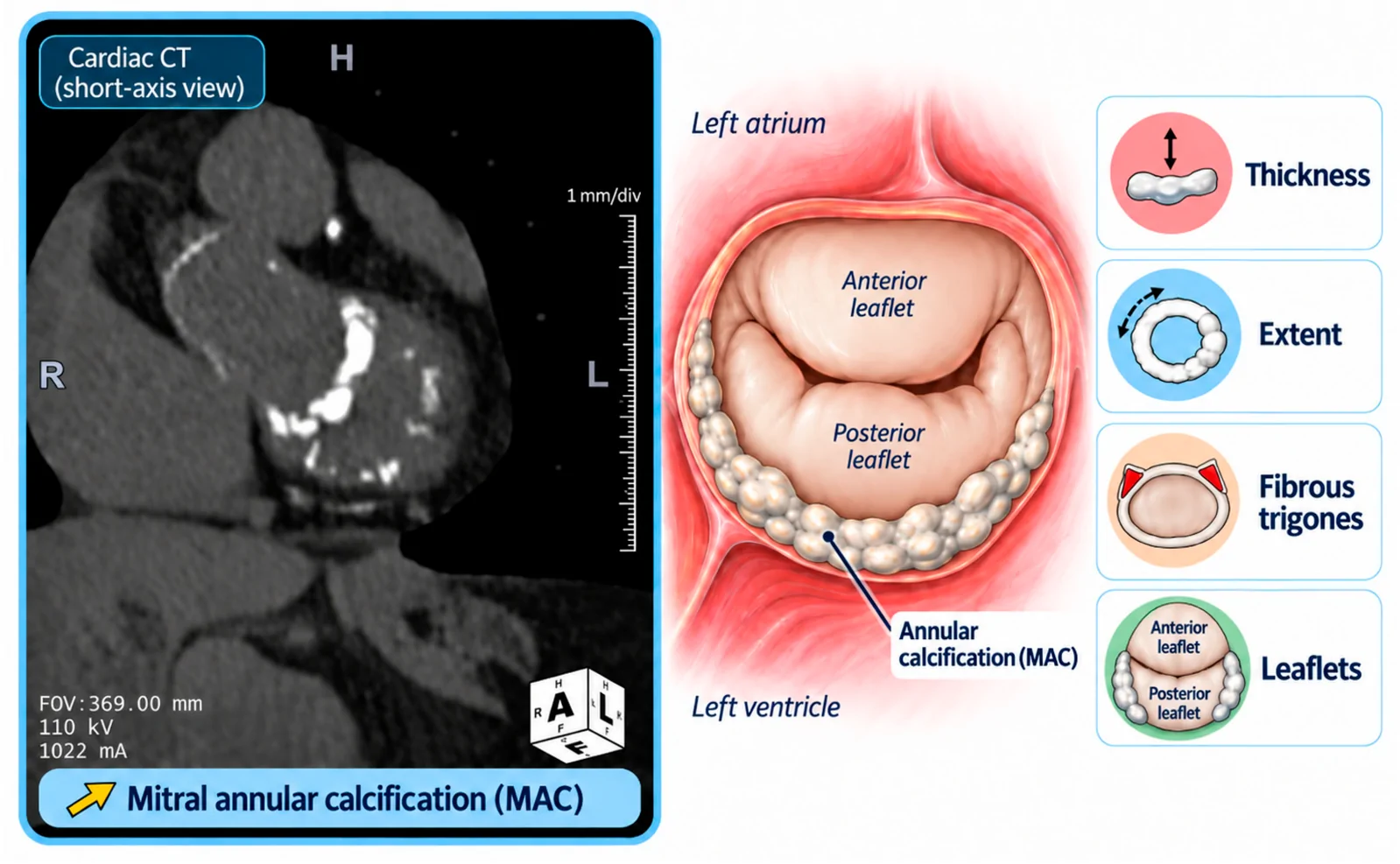
CT assessment of mitral annular calcification (MAC). Left: cardiac CT short-axis view showing extensive calcification of the mitral annulus. Center and right: schematic representation of CT-based MAC assessment, including calcium thickness, circumferential extent, involvement of the fibrous trigones, and extension to the mitral leaflets, as incorporated in the Guerrero MAC severity score.

**Figure 13:**
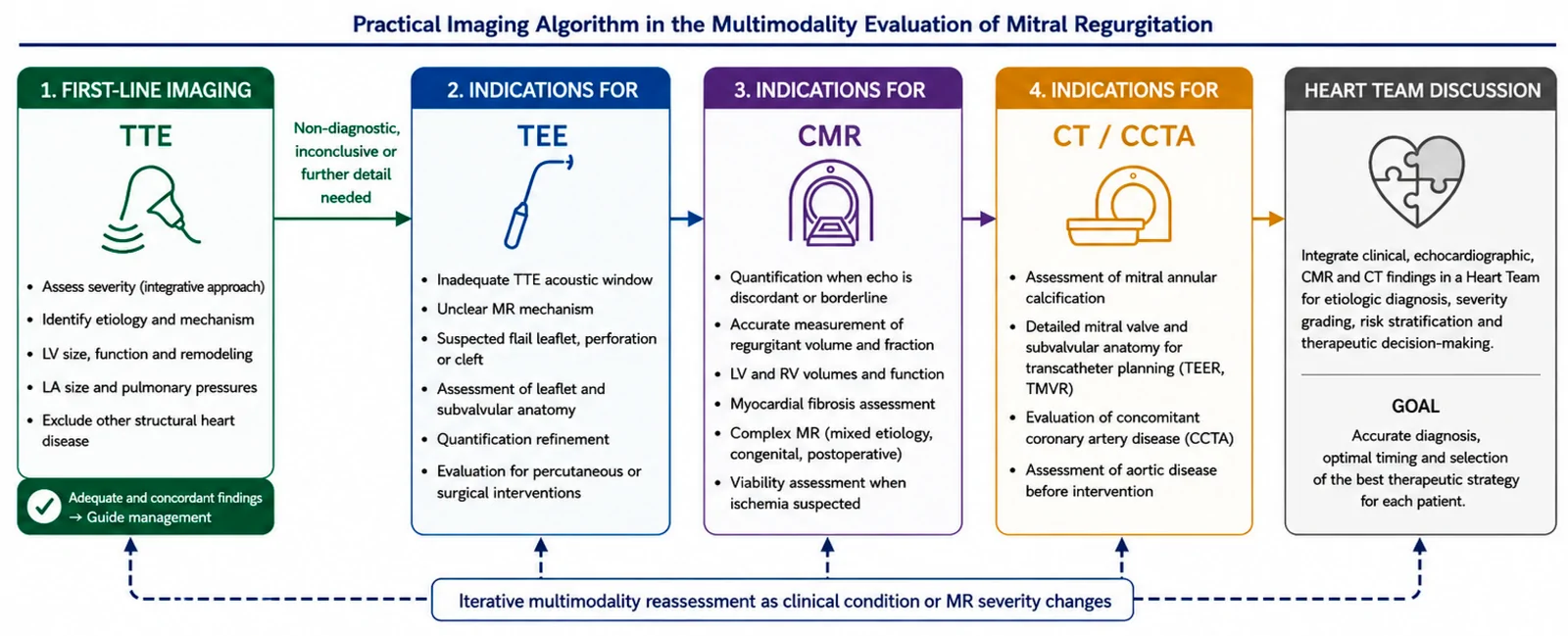
Practical multimodality imaging algorithm for the evaluation of mitral regurgitation (MR). The figure illustrates a multimodality imaging algorithm for the evaluation of MR. TTE represents the first-line imaging modality for the assessment of the MR mechanism, severity, hemodynamic consequences, and cardiac remodeling. TEE is performed when TTE provides suboptimal or inconclusive information, or when detailed anatomical characterization and a procedural assessment are required. CMR may be considered when echocardiographic findings remain discordant or inconclusive, providing a complementary quantitative assessment of the regurgitant volume and regurgitant fraction, as well as myocardial and ventricular characterization. CT is reserved for selected patients when additional anatomical information is required, particularly for the assessment of mitral annular calcification, complex anatomy, and planning of transcatheter mitral valve replacement. The multimodality imaging findings are ultimately integrated within the clinical context and discussed by the Heart Team to guide individualized therapeutic decision-making. MR: mitral regurgitation; TTE: transthoracic echocardiography; TEE: transesophageal echocardiography; CMR: cardiac magnetic resonance; CT: computed tomography.

**Table 1:** Echocardiographic methods and parameters for mitral regurgitation quantification: advantages and limitations.

Parameter	Advantages	Limitations
**Color Flow Doppler**	Rapid and intuitive; useful for identifying origin and mechanism of MR; widely available.	Highly dependent on machine settings, jet direction, and pressure gradient; inaccurate in eccentric jets (“splay” artifact); not suitable as a standalone method.
**Vena Contracta (VC)**	Simple, relatively reproducible; good surrogate of regurgitant orifice in central jets.	Assumes circular orifice; limited accuracy in eccentric and multiple jets (should not be summed); 2D measurement may underestimate complex orifice geometry.
**CW Doppler Density and Contour**	High signal density correlates with more severe MR; triangular early-peaking contour suggests elevated LA pressure.	Qualitative and operator-dependent; influenced by gain settings and alignment.
**Mitral Inflow (E-wave velocity)**	Easy and fast to obtain; reflects increased transmitral flow in significant MR; useful as a supportive parameter.	Non-specific; influenced by LV relaxation, filling pressures, heart rate, and rhythm; not reliable as a standalone marker.
**Pulmonary Venous Doppler**	Systolic blunting or flow reversal is highly specific for severe MR; provides hemodynamic confirmation of severity.	Technically challenging; depends on sampling position; may be unreliable in atrial fibrillation or elevated LA pressure from other causes; limited feasibility in some patients.
**PISA (Proximal Isovelocity Surface Area)**	Well-validated quantitative method; direct estimation of regurgitant lesion severity	Limited accuracy in eccentric or multiple jets; sensitive to image quality and Doppler alignment; PISA radius varies during systole;
**Volumetric Method**	Geometry-independent; particularly useful in FMR.	Sensitive to errors in LV volumes and LVOT measurements; cumulative measurement error; affected by arrhythmias, multivalvular diseases, and poor image quality.
**Mitral-to-Aortic VTI Ratio (PW Doppler)**	Simple, fast, and reproducible; helpful adjunct when other methods are inconclusive.	Only valid in absence of mitral stenosis or significant aortic valve disease; affected by loading conditions; indirect measure, not for standalone grading.

**Table 2:** Comparison of guideline-based echocardiographic and proposed/outcome-informed CMR thresholds for MR severity.

MR Phenotype	Modality/Parameter	Mild	Moderate	Severe	Evidence/Interpretation
**Primary MR**	Echo EROA RVol RF	<20 mm^2^ <30 mL <30%	20–39 mm^2^ 30–59 mL 30–49%	≥40 mm^2^ ≥60 mL ≥50%	Guideline-based, integrated ASE/EACVI assessment [14,37]
**Primary MR**	CMR RVol RF	<30 mL <20%	30–55 mL 20–39%	>55–60 mL >40%	Proposed CMR categories [38]; outcome threshold > 55 mL/>40% [39]; RVol ≥ 60 mL used in [19]
**Secondary MR**	Echo EROA RVol RF	<20 mm^2^ <30 mL <30%	20–39 mm^2^ 30–59 mL 30–49%	≥40 mm^2^ * ≥60 mL † ≥50%	Guideline-based, integrated ASE/EACVI assessment [14,37]
**Secondary MR**	CMR Rvol § RF	<30 mL <20%	30–55 mL 20–34%	>55–60 mL ≥35%	Proposed categories [38]; RF ≥ 20%/≥35% outcome-based [40]; risk modified by scar [41]

* In secondary MR, EROA ≥ 30 mm^2^ may support severe MR when the regurgitant orifice is elliptical. † RVol ≥ 45 mL may support severe MR in low-flow conditions. § The secondary-MR CMR RVol categories are reproduced from the contemporary EHJ review [38], but the principal outcome studies supporting severity thresholds predominantly validated RF rather than RVol [40,41]. All echocardiographic thresholds must be interpreted using an integrated multiparametric approach; CMR thresholds are not interchangeable with echocardiographic guideline cut-offs.

**Table 3:** Comparison between 64-section CT and MR imaging measurements in 49 patients.

Parameter	64-Section CT	MR Imaging	*p* Value *	Pearson Coefficient †
End-diastolic volume (mL)	191.4 ± 75.2	185.1 ± 70.3	0.13	0.93 (0.87, 0.95)
End-systolic volume (mL)	92.2 ± 45.7	91.2 ± 40.4	0.72	0.92 (0.86, 0.95)
Ejection fraction (%)	51.9 ± 10.5	50.5 ± 10.1	0.07	0.88 (0.78, 0.92)
Left ventricular stroke volume (mL)	98.7 ± 43.7	93.6 ± 42.3	0.16	0.91 (0.85, 0.95)
Right ventricular stroke volume (mL)	52.8 ± 34.3	48.9 ± 37.0	0.13	0.88 (0.80, 0.93)
RV (mL)	45.0 ± 22.7	46.3 ± 27.1	0.56	0.89 (0.81, 0.94)
RF (%)	39.2 ± 18.5	38.7 ± 17.2	0.87	0.91 (0.84, 0.95)

Note—unless otherwise indicated, data are means ± standard deviations. * Paired *t* test. † Data in parentheses are 95% CIs.

## Data Availability

No new data were created or analyzed in this study. Data sharing is not applicable to this article.

## Abbreviations

The following abbreviations are used in this manuscript:

aFMR	Atrial Functional Mitral regurgitation
AR	Aortic regurgitation
AROA	Anatomical regurgitant orifice area
BSA	Body surface area
CT	Computed tomography
CMR	Cardiac magnetic resonance
CW	Continuous wave Doppler
ECG	Electrocardiography
EROA	Effective regurgitant orifice area
FMR	Functional mitral regurgitation
HF	Heart failure
IC	Inter-commissural
LA	Left atrium
LAVI	Left atrial volume index
LUPV	Left upper pulmonary vein
LV	Left ventricle (LV)
LVEDV	Left ventricular end-diastolic volume
LVESV	Left ventricular end-systolic volume
LVOT	Left ventricular outflow tract
MA	Mitral annulus
MAC	Mitral annular calcification
ME	Mid-esophageal
MIP	Maximum intensity projection
MPR	Multiplanar reformation
MV	Mitral valve
MVP	Mitral valve prolapse
NYHA	New York Heart Association
PMR	Primary mitral regurgitation
PISA	Proximal isovelocity surface area
PRF	Pulse repetition frequency
PW	Pulsed wave Doppler
RVol	Regurgitant volume
RF	Regurgitant fraction
RUPV	Right upper pulmonary vein
RV	Right ventricle
RVEDV	Right ventricular end diastolic volume
SPAP	Systolic pulmonary artery pressure
TEE	Trans-esophageal echocardiography
TEER	Transcatheter edge-to-edge repair
TMVR	Transcatheter mitral valve replacement
TSV	Total stroke volume
TTE	Trans-thoracic echocardiography
vFMR	Ventricular functional mitral regurgitation
VC	Vena contracta
VENC	Velocity encoding
VRT	Volume rendering technique (VRT)
VTI	Velocity–time integral
